# Global cellular proteo-lipidomic profiling of diverse lysosomal storage disease mutants using nMOST

**DOI:** 10.1126/sciadv.adu5787

**Published:** 2025-01-22

**Authors:** Felix Kraus, Yuchen He, Sharan Swarup, Katherine A. Overmyer, Yizhi Jiang, Johann Brenner, Cristina Capitanio, Anna Bieber, Annie Jen, Nicole M. Nightingale, Benton J. Anderson, Chan Lee, Joao A. Paulo, Ian R. Smith, Jürgen M. Plitzko, Steven P. Gygi, Brenda A. Schulman, Florian Wilfling, Joshua J. Coon, J. Wade Harper

**Affiliations:** ^1^ Department of Cell Biology, Blavatnik Institute, Harvard Medical School, Boston, MA 02115, USA.; ^2^ Aligning Science Across Parkinson’s (ASAP) Collaborative Research Network, Chevy Chase, MD 20815, USA.; ^3^ Department of Biomolecular Chemistry, University of Wisconsin–Madison, Madison, WI 53706, USA.; ^4^ Morgridge Institute for Research, Madison, WI 53715, USA.; ^5^ Department of Molecular Machines and Signaling, Max Planck Institute of Biochemistry, Martinsried, Germany.; ^6^ Mechanisms of Cellular Quality Control, Max Planck Institute of Biophysics, Frankfurt, Germany.; ^7^ CryoEM Technology, Max Planck Institute of Biochemistry, Munich, Germany.; ^8^ Department of Chemistry, University of Wisconsin–Madison, Madison, WI 53706, USA.

## Abstract

Lysosomal storage diseases (LSDs) comprise ~50 monogenic disorders marked by the buildup of cellular material in lysosomes, yet systematic global molecular phenotyping of proteins and lipids is lacking. We present a nanoflow-based multiomic single-shot technology (nMOST) workflow that quantifies HeLa cell proteomes and lipidomes from over two dozen LSD mutants. Global cross-correlation analysis between lipids and proteins identified autophagy defects, notably the accumulation of ferritinophagy substrates and receptors, especially in *NPC1*^−/−^ and *NPC2*^−/−^ mutants, where lysosomes accumulate cholesterol. Autophagic and endocytic cargo delivery failures correlated with elevated lysophosphatidylcholine species and multilamellar structures visualized by cryo–electron tomography. Loss of mitochondrial cristae, MICOS complex components, and OXPHOS components rich in iron-sulfur cluster proteins in *NPC2*^−/−^ cells was largely alleviated when iron was provided through the transferrin system. This study reveals how lysosomal dysfunction affects mitochondrial homeostasis and underscores nMOST as a valuable discovery tool for identifying molecular phenotypes across LSDs.

## Teaser

A nanoflow multiomic workflow reveals how lysosomal dysfunction disrupts autophagy and mitochondrial homeostasis in LSD mutants.

## INTRODUCTION

Lysosomes are the central organelle for degradative and recycling functions within eukaryotic cells, and digest cargo (e.g., macromolecules) delivered via various trafficking systems, including autophagy or endocytosis (*1*, *2*). Moreover, lysosomes are integral to lipid catabolism and nutrient sensing via the MTOR protein kinase. Genetic and clinical studies have linked lysosomal dysfunction to a wide variety of storage accumulation pathologies, called “lysosomal storage diseases” or henceforth LSDs (*3*). More than 50 LSDs have been described and while individually rare, their combined prevalence is 1:5000 in live births, while some population groups carry higher incidence rates. As expected, given the central role of lysosomes in cellular health, LSDs have been linked to various human diseases, including Niemann-Pick type C1/2 (*NPC1* and *NPC2*), Gaucher (*GBA1*), Pompe (*GAA*), Danon (*LAMP2*), and neuronal ceroid lipofuscinoses (*GRN*), and increased risk of Parkinson’s disease is observed within a subset of LSDs (*SMPD1*, *ASAH1*, *ATP13A2*, *CTSD*, and *GBA1*) (*4*–*8*). The majority of LSD genes encode catabolic enzymes (e.g., hydrolases) that functions in the lysosomal lumen, although an important subset function as small molecule/ion transporters that maintain metabolic balance within the lysosome. Defects in these activities can result in a cascade of effects on downstream processes, leading to the accumulation of various types of materials: sphingolipids, mucopolysaccharides, glycoproteins, and lipofuscin (*3*). Additional storage material can also accumulate as a secondary response to the primary storage defect, including phospholipids, glycosphingolipids, and cholesterol. Several LSDs also lead to an imbalance in lipid catabolism and/or defects in sphingolipid metabolism (e.g., *CLN5* and *GRN*) (*9*, *10*), raising the question of the extent to which dysregulation of lipid homeostasis underlies divergent LSDs (*11*).

Understanding complex relationships across lipidomes and proteomes necessitates an approach for molecular profiling across the diversity of LSD loss-of-function mutations. In principle, systematic profiling should facilitate the identification of similarities and differences in molecular phenotypes, defined here as alterations in the abundance of proteins and lipids, exhibited across the various disease classes. Here, we develop a highly sensitive nanoflow liquid chromatography (LC) multi-omic single-shot technology (nMOST) for simultaneous analysis of lipids and proteins. nMOST represents a second-generation implementation of the microflow LC MOST method (μMOST) (*12*) and integrates a multiomic sample preparation strategy (*13*) with intelligent lipidomic data acquisition (*9*, *14*) to provide substantially improved sensitivity. To demonstrate the generality of the approach and to create a resource for the community, we applied nMOST to a collection of more than two dozen LSD mutant HeLa cell lines. Global lipid-protein cross-correlation analysis reveals patterns of accumulation of individual proteins and lipids in distinct genotypes. Among the strongest correlations identified was autophagy and ferritinophagy signatures associated with *NPC1*^−/−^ and *NPC2*^−/−^ cells, consistent with previous studies (*15*–*17*), but unexpectedly, we also observed defects in the mitochondrial proteome, particularly in *NPC2*^−/−^ cells. *NPC1* and *NPC2*, which are mutated in Niemann-Pick disease, play a key role in cholesterol trafficking out of the lysosome (*18*). Cholesterol esters (CEs) within the lysosomal lumen are hydrolyzed by the LSD-associated protein LIPA (lipase A), and the cholesterol product is then carried by the lumenal NPC2 protein to the membrane-embedded NPC1 transporter, facilitating cholesterol egress from the lysosome. In addition to the known accumulation of cholesterol in cells lacking *NPC1* or *NPC2*, nMOST analysis additionally revealed alterations in other lipids including lyso–phosphocholine (PC) species, which are major membrane building blocks in cells.

Through a series of cell biological validation experiments that demonstrate the value of this approach, we demonstrate in *NPC2*^−/−^ cells that defects in autophagic turnover of ferritin—a major mechanism for cellular control of iron availability (*19*, *20*)—lead to defects in mitochondrial cristae and oxidative phosphorylation (OXPHOS) complexes rich in iron-sulfur cluster proteins, which can be ameliorated by supplying iron through the transferrin system. Iron similarly rescued the abundance of OXPHOS machinery during differentiation of *NPC1^−/−^* or *NPC2^−/−^* mutant stem cells to induced neurons (iNeurons). Moreover, we demonstrate a defect in delivery of autophagic and endocytic cargo to the lysosomal lumen. These defects correlate with the formation multilamellar structures within lysosomes, as visualized at nanometer resolution using cryo–electron tomography (cryo-ET), and with accumulation of lyso-PC as a possible membrane building block. This resource of quantitative lipidomic and proteomic data across diverse classes of LSD mutations—available through an online data portal (https://coonlabdatadev.com/, for access see data and materials availability statement)—provides a rich landscape for further biological discovery.

## RESULTS

### Robust proteomic and lipidomic analysis using nMOST

**Fig. 1.**
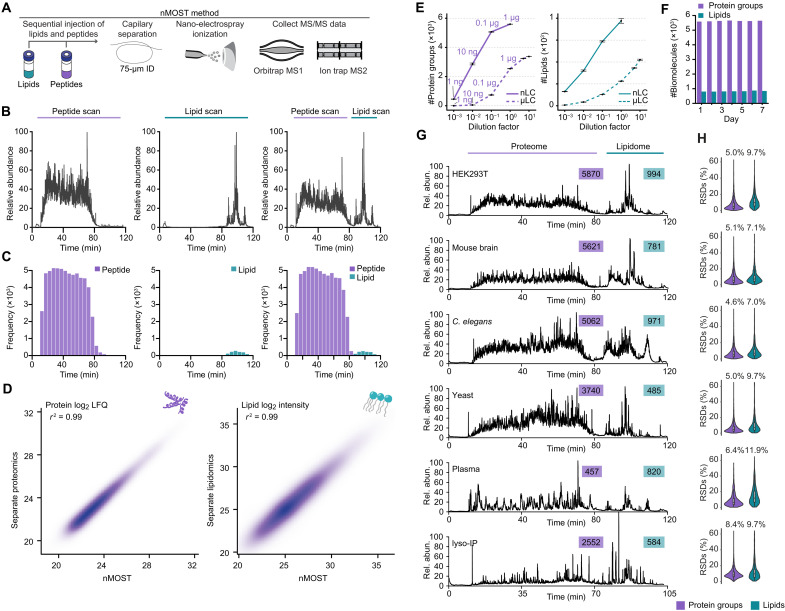
Development and benchmarking of nMOST for proteomic and lipidomic analysis. (**A**) Schematic of the nMOST method, which allows proteome and lipidome analysis by LC-MS. Lipid and protein extracts isolated from the same cell sources are sequentially injected onto LC before elution with an organic gradient and MS analysis (see Methods). (**B**) Chromatograms showing HEK293 cell peptide and lipid elution features during a 120-min gradient examining (left) total protein extract, (middle) total lipid extract, and (right) sequentially loaded protein and lipid extracts and nMOST analysis. The vast majority of peptides elute before 80 min, while the majority of lipids elute between 80 and 120 min. (**C**) Peptide and lipid identifications from the corresponding LC-MS run in (B). (**D**) Correlation of proteins (left) and lipids (right) identified by separate LC-MS (*y* axis) versus nMOST (*x* axis). *r*^2^ values are > 0.99. (**E**) Number of protein groups and lipid groups identified by nMOST versus μMOST methods. nMOST routinely outperformed μMOST for both proteins (left) and lipids (right). Amount of peptide injections are labeled above the line for each nMOST and μMOST. (**F**) Performance was comparable for both proteins and lipids when measured daily over a 7-day acquisition period. (**G**) nMOST allows simultaneous analysis of proteins and lipids from HEK293 cells, mouse brain extracts, *C. elegans* extracts, budding yeast extracts, human plasma, and lysosomes from HeLa cells isolated by lyso-IP. (**H**) RSD values for the data in (G).

We previously reported μMOST as an approach to acquire proteomic and lipidomic data simultaneously (*12*). However, the sensitivity of microflow was limited, which prompted the development of an analogous nanoflow method with substantially increased sensitivity (Fig. 1A). nMOST takes advantage of the fact that the vast majority of lipid species elute from reverse-phase columns well after the vast majority of peptides, as indicated when peptide and lipid extracts from human embryonic kidney (HEK) 293 cells are analyzed separately with the same mobile phase gradient (Fig. 1, B and C, left and middle). We found, however, that sequential loading of lipid and peptide extracts followed by LC–mass spectrometry (LC-MS) provided virtually identical performance (Fig. 1, B and C, right), with correlation coefficients (*r*) for both protein label-free quantification (LFQ) and lipid intensity > 0.99 (Fig. 1D and fig. S1A). Thus, the presence of peptides on the immobile phase did not affect lipid detection or quantification and vice versa. Consistent with added sensitivity of the nanoflow approach, nMOST delivered >2-fold more protein (5593 versus 2540) or > 3-fold more lipid (967 versus 281) identifications as compared with μMOST when analyzing 1 μg of HEK293 cell-derived peptides or 0.03% lipid mass in a single LC-MS run (Fig. 1E). Direct comparison between nMOST and μMOST in quantifying protein groups of sub-cellular organelles (mitochondria, lysosomes, endosomes, and nucleus) across three magnitudes of sample dilution demonstrates the superiority of nMOST over previous implementations (Fig. 1E and fig. S1B). Moreover, the method was found to be robust, with a similar number of biomolecules quantified over an extended period of data collection, with a median relative standard deviation (RSD) of 5.0% for proteins and 13.2% for lipids [Fig. 1F and fig. S1 (C and D), and see below].

To demonstrate versatility, we benchmarked nMOST performance across multiple species (HEK293 cells, mouse brain, *Caenorhabditis elegans*, and budding yeast) and sample types (cell extracts, plasma, and purified lysosomes) (Fig. 1G). We observed the expected complexity of proteomes across the various samples and routinely detected thousands of proteins and ~500 to 1000 lipid species (Fig. 1G), with RSDs of 4.6 to 8.4% for proteins and 7.0 to 11.9% for lipids (Fig. 1H). The consistent identifications and stable quantifications over extended periods of analysis time highlight the robustness of the method and reinforce its potential for reproducible and high-quality data acquisition to elucidate complex relationships between proteomes and lipidomes.

### A tool kit for systematic analysis of LSD genes

**Fig. 2.**
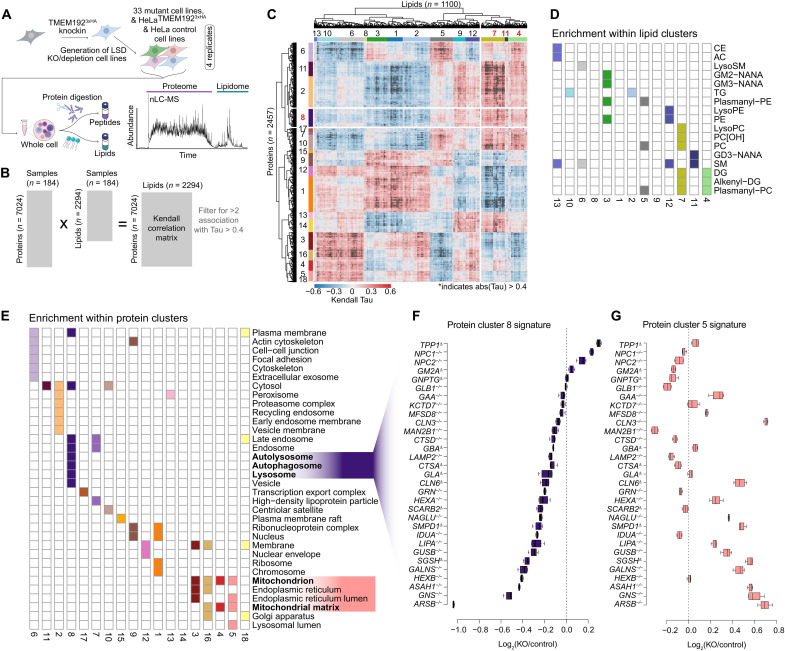
Landscape of total proteomes and lipidomes from LSD mutant cells using nMOST. (**A**) Schematic describing the method for analysis of total cell extracts across 33 LSD mutants. Protein and lipid extracts were isolated from the samples in quadruplicate and then sequentially injected for analysis by LC-MS over a 120-min gradient. (**B**) Schematic depicting the method used for lipid/protein cross-correlation analysis using a Kendall rank correlation (filtered for >1 association with Tau > 0.4). (**C**) Heatmap for Tau values. Clusters for proteins and lipids are shown. (**D**) Schematic showing the enrichment of specific lipids within individual lipid clusters. (**E**) Schematic showing the subset of GO term cellular compartment enriched within individual protein clusters. (**F**) Summed protein cluster 8 signature [sum abundance of all proteins within cluster 8 (enriched for autophagy terms)] across the LSD mutant cells plotted as log_2_FC [KO/wild type (WT)]. (**G**) Signature of protein cluster 5 (sum protein abundance relative to WT) across the LSD mutant cells.

The data described so far points to the utility of the nMOST approach in quantifying alterations in lipids and proteins in a diverse spectrum of biological samples. To demonstrate the utility of this approach, we set out to generate molecular landscapes of cells that are deficient in various LSD proteins. We used CRISPR-Cas9 to attempt targeting of 52 LSD genes in HeLa cell lines containing endogenously tagged TMEM192-HA for lysosome detection (HeLa^TMEM192-HA^) (Fig. 2A, fig. S1E, and table S1) (*21*, *22*). In total, we validated a total of 38 mutants across multiple functional classes of LSDs using a combination of sequencing and proteomic approaches: 23 homozygous deletions, 5 heterozygous deletions, and 10 mutants containing one or more alleles with an in-frame deletion (fig. S1F and table S1). We applied nMOST to total cell extracts using quadruplicate independent cultures (Fig. 2A). This was accomplished by running the samples across 15 sets, where each set contains multiple HeLa^TMEM192-HA^ or control parental samples and were flanked by instrument quality control (QC) runs, ensuring stable performance. In total, 318 whole-cell extract samples were subjected to nMOST, representing 4 weeks of cumulative continuous data collection, with little change in method performance, as indicated by the log_2_ abundance values for proteins and lipids. In addition, 45 QC samples spread throughout the data collection period demonstrated high-performance reproducibility (fig. S1, G to I). In total, more than 7000 proteins and 2000 lipids were routinely identified and quantified in whole-cell extracts (table S2). In the majority of cases for heterozygous and in-frame deletion clones, the levels of target proteins detected by proteomics, when detected, indicated substantially reduced protein levels (fig. S1F; see table S1 for detailed genotyping results of each clone). We excluded heterozygous deletions in the subsequent analysis, leaving 33 LSD mutant cell lines which serve as a resource for phenotypic analysis of a broad range of LSD genes.

### Molecular fingerprinting of LSDs using nMOST

To identify molecular fingerprints across the LSD mutant cells, we performed lipid-protein cross-correlation analysis using a Kendall rank approach (Fig. 2B), resulting in 1100 lipids and 2457 proteins with at least two correlations with |Tau| > 0.4. Hierarchical clustering of the correlation matrix revealed 13 lipid and 18 protein clusters (Fig. 2C and table S3) with significant enrichment of either lipid classes or subcellular compartments across the proteo-lipidomic landscape for 33 LSD mutant cell lines (Fig. 2, D and E). Given the importance of autophagy in cellular homeostasis, we focused on protein cluster 8; this cluster encompassing lysosome, autolysosome, and autophagosome terms and correlated significantly with PCs, lyso-PCs, plasmanyl-PCs, diacylglycerols (DGs), alkenyl-DGs, and gangliosides in lipid clusters 4, 7, and 11 (Fig. 2, C to E). The summed cluster 8 signature plotted as log_2_[knockout (KO)/control] indicated that *NPC1^−/−^*, *NPC2^−/−^*, and *TPP1*^−/−^ cells were among the strongest candidates for affected mutants within the lysosome cluster, cluster 8 (Fig. 2F and tables S2 and S3). This contrasted with a variety of other LSD mutants, which were enriched in clusters 4 and 5 and displayed increased levels of mitochondrial proteins (e.g., *LIPA^−/−^*, *ARSB^−/−^*, and *GNS^−/−^*) (Fig. 2G).

To further deconvolute which organelles and processes were most affected by altered cholesterol efflux from lysosomes, we created a curated sublist of organelle proteins (1784 IDs; see Materials and Methods), encompassing annotated proteins for mitochondria, lysosome, endosome, Golgi, endoplasmic reticulum, and proteasome, as well as autophagy and iron homeostasis, and performed *k*-means clustering (fig. S1J; contains average abundance of indicated annotation group). *NPC1^−/−^* and *NPC2^−/−^* clustered closely together (genotype group 2), while *LIPA^−/−^* was located in group 5, consistent with the differential effect on organelle proteomes (fig. S1J). We focused on three clusters of interest: While proteins belonging to *k*-means cluster 3 [Gene Ontology (GO): (regulation of) mitochondrial RNA catabolic process] were increased across all three genotypes, cluster 4 [GO: (macro-) autophagy and vacuole organization] was elevated in group 2 mutants containing *NPC1*^−/−^ and *NPC2*^−/−^, but not in *LIPA^−/−^* in group 5, while *k*-means cluster 5 (GO: adenosine triphosphate synthesis and aerobic electron transport chain) was increased in group 5 but not group 2 (fig. S1J). Thus, the signatures observed in Fig. 2 (C to E) for *NPC* mutants and *LIPA^−/−^* may reflect changes in organelle homeostasis, especially mitochondria and autophagy.

### A nMOST-LSD resource for lipid-protein correlation analysis

**Fig. 3.**
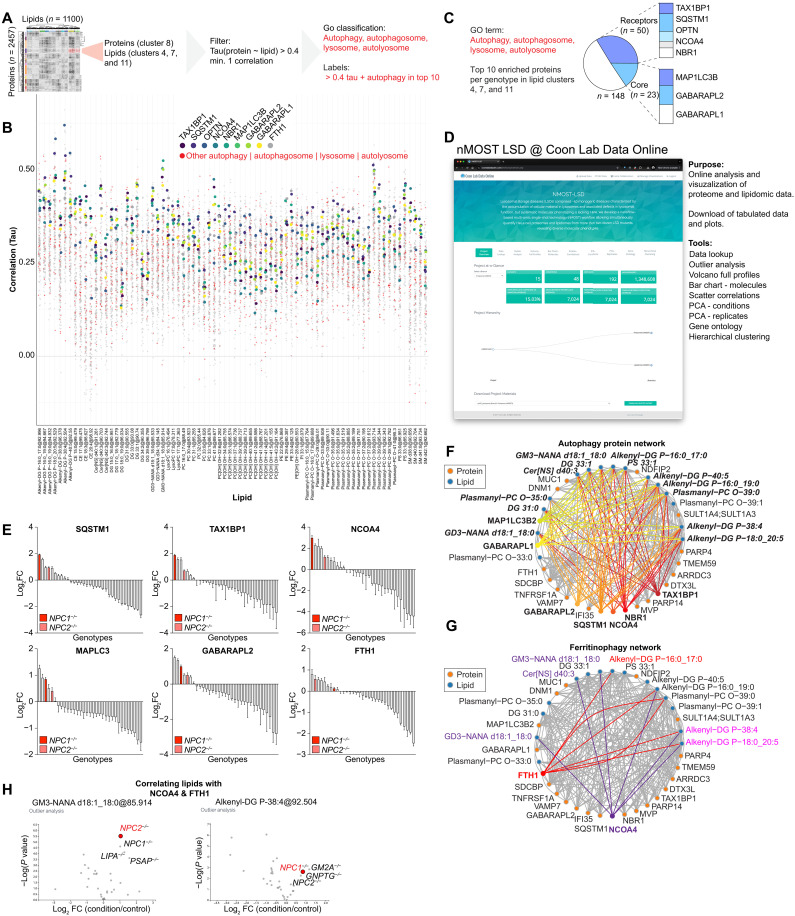
An nMOST-LSD resource for lipid-protein correlation analysis. (**A**) Schematic of search strategy to find functional protein-lipid relationships from LSD-nMOST cross-ome dataset. (**B**) Manhattan-style plot of lipid species (*x* axis, lipid clusters 4, 7, and 11) versus protein correlations from protein cluster 8. Red dots represent proteins associated with GO terms autophagy, autophagosome, lysosome, and autolysosome. In addition, select autophagy proteins are highlighted in viridis color scheme. (**C**) Pie chart of top 10 enriched proteins from (B). Autophagy receptors and autophagy core components represent ~50 of the hits, and composition is shown on the right. (**D**) Screenshot of nMOST LSD on the Coon Lab Data Online portal. Tools available online are listed on the right. (**E**) Log_2_FC ranked bar graph of indicated autophagy proteins across all analyzed LSD genotypes. *NPC1^−/−^* and *NPC2^−/−^* genotypes are highlighted in shades of red. Data extracted from online portal. (**F** and **G**) Protein-lipid network extracted from protein cluster 8 and lipid clusters 4, 7, and 11. (F) Protein-lipid connections for autophagy markers. (G) Protein-lipid connections for ferritinophagy markers. (**H**) Outlier analysis of two lipid species highlight correlated with NCOA4 and FTH1 [see (G)]. Data were extracted from online portal.

The availability of deep proteome and lipidome data across cells lacking various LSD proteins provides an opportunity to identify common and distinct patterns, potentially identifying specific “molecular phenotypes” that may contribute to distinct underlying cellular defects. An example of how such correlations can be used is provided in Fig. 3, in this case examining lipid-protein correlations for protein cluster 8 and the corresponding lipid clusters 4, 7, and 11 (Fig. 3, A and B, also see Fig. 2C). We plotted all lipid-protein pairs with at least one correlation with Tau > 0.4, performed GO enrichment analysis, and highlighted autophagy proteins of interest (red dots). Proteins in cluster 8 had overall high correlations to the select lipid species, which was especially true for a subset of autophagy receptors. We next examined the top 10 enriched proteins per lipid species and stratified them by their frequency (Fig. 3C). Almost half of the top 10 proteins were either ubiquitin-binding autophagy receptors, nuclear receptor coactivator 4 (NCOA4), or autophagy-related protein 8 (ATG8) proteins.

To facilitate further mining of these data by the community, we established an online portal, where the LSD-nMOST data can be parsed, analyzed, and visualized in multiple ways (Fig. 3D). This portal allows visualization of abundance profiles for individual proteins across all of the LSD mutant cell line proteomes, as shown for several autophagy receptors, and highlighting the finding that cells lacking NPC1 or NPC2 typically exhibit among the highest levels of these receptors, especially the ferritinophagy receptor NCOA4 and one of its main cargo, ferritin (FTH1) (Fig. 3E). Network analysis for general autophagy markers (Fig. 3F) or ferritinophagy (Fig. 3G) revealed correlations involving specific lipids in distinct lipid classes, including GABARAPL1, and ubiquitin-binding autophagy receptors with alkenyl-DG, GM3, Cer[NS], and DG3 species (Fig. 3F). Linkage between multiple ubiquitin-binding autophagy receptors and these specific lipids may provide a lipid fingerprint associated with accumulation of specific receptors. Similarly, the portal allows the identification of mutant cell lines that display significantly different patterns of specific biomolecules (on both protein and lipid levels) compared to the rest of the LSD-nMOST dataset and which other genotypes display a similar pattern (outlier analysis). For example, *NPC1*^−/−^ and *NPC2*^−/−^ display strong correlations between the NCOA4/FTH1 pair and alkenyl-DG or GM3 species (Fig. 3, F and G), and several other genotypes showed similar lipid outlier profiles (Fig. 3H).

### nMOST analysis of lysosomal cholesterol pathway mutants

The data described above highlighted the link between autophagy signatures and LSD proteins linked with the lysosomal cholesterol transport pathway. To examine these relationships in greater detail, we performed validation experiments in cells lacking *NPC1*, *NPC2*, or *LIPA*—as well as GAA, a glycogen storage mutant linked with Pompe’s disease (fig. S2A and table S1). As expected, lysosomes of *NPC1*^−/−^ and *NPC2*^−/−^ mutants, but not *LIPA*^−/−^ or *GAA*^−/−^ mutants, markedly accumulate cholesterol within lysosomes based on staining with the cholesterol binding probe Filipin (fig. S2, A and B). Lysosomes in *NPC1*^−/−^ and *NPC2*^−/−^ cells displayed slightly elevated pH (5.9 and 6.5, respectively, compared with *LIPA*^−/−^, *GAA*^−/−^, and control cells, with pH of ~5.4 to 5.7) in line with previous reports (fig. S2C) (*23*).

We performed nMOST analysis of this “4KO” cohort in quadruplicate replicates under both full media (fed) and nutrient stress conditions know to induce autophagy [Earle’s balanced salt solution (EBSS), 6 hours] (fig. S2D and table S4). The absence of the deletion target was verified by LFQ of nMOST data (fig. S2E), and principal components analysis (PCA) revealed high sample and treatment reproducibility (fig. S2, F and G), pointing to the robustness of the nMOST method. Hierarchical clustering of the 1007 lipids quantified revealed distinct alterations in the abundance of multiple lipid classes, with *NPC1*^−/−^ and *NPC2*^−/−^ cells clustering together, as anticipated (fig. S2H). Individual lipid species within specific classes demonstrates divergent patterns of accumulation or loss for individual genotypes (e.g., PC species in *LIPA*^−/−^ or *NPC1*^−/−^ cells) (fig. S2H). As explored below, we identified a set of lyso-PC species that were selectively increased in *NPC1*^−/−^ and *NPC2*^−/−^ cells in both fed and starved conditions (fig. S2H). Similarly, hierarchical clustering of proteomic data focused on the major cellular organelle systems with links to autophagy and mitochondrial pathways (see Materials and Methods for description of curated organelle proteomes) revealed distinct patterns of alterations in protein abundance based on genotype, with *NPC1*^−/−^ and *NPC2*^−/−^ cells clustering together with *LIPA*^−/−^ (fig. S2I). By comparison, *GAA*^−/−^ cells had fewer alterations in protein abundance relative to control cells (fig. S2I).

**Fig. 4.**
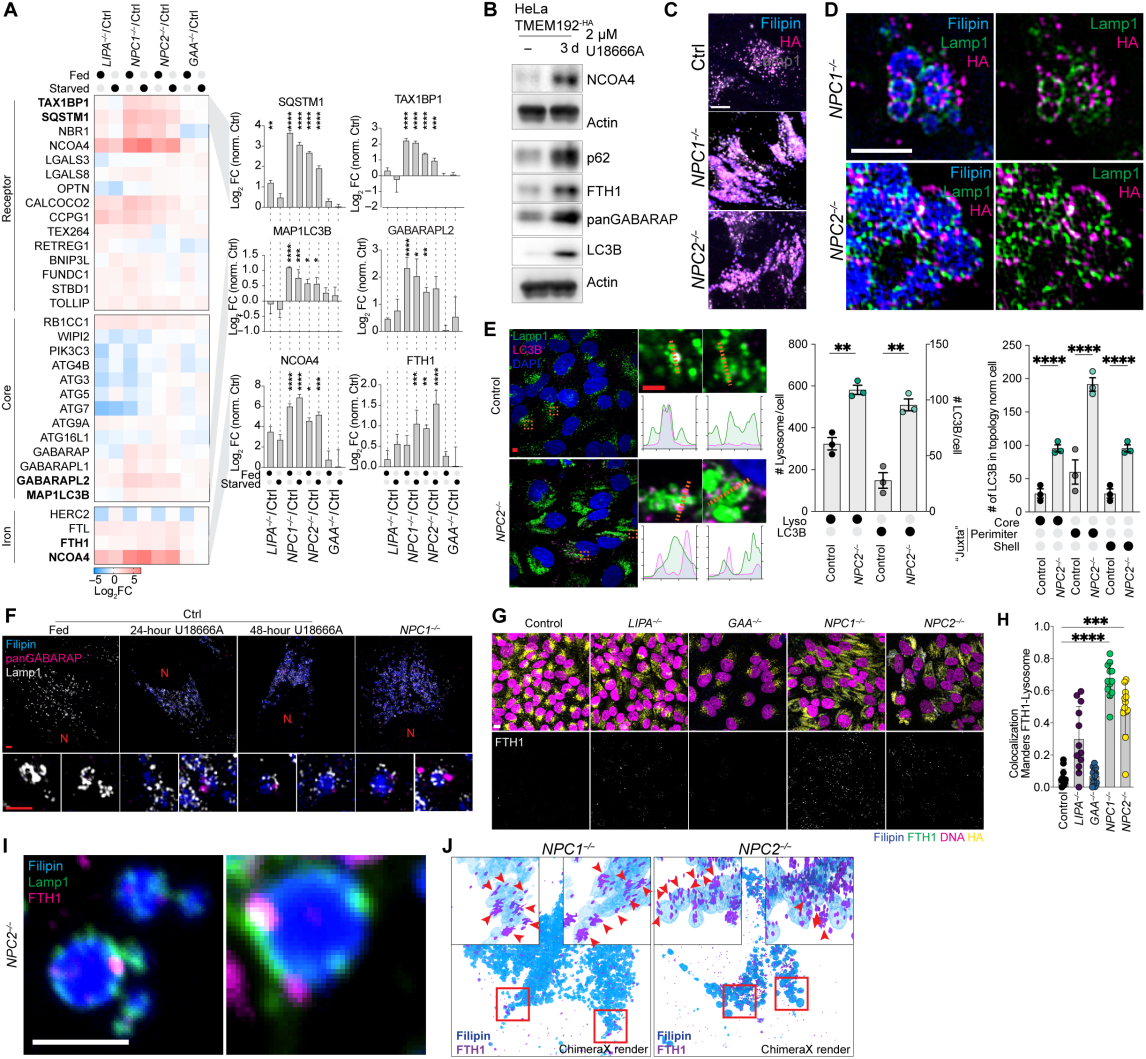
Juxta-lysosomal autophagy receptors and ferritin accumulation in *NPC1*^−/−^ and *NPC2*^−/−^ cells. (**A**) Log_2_FC relative to control cells for the indicated autophagy receptors for 4KO cells. MAPLC3B: ****P < 0.0001, ***P = 0.0001, and *P = 0.0129 and 0.0157. SQSTM1: ****P < 0.0001 and **P = 0.0047. TAX1BP1: ****P < 0.0001 and ***P = 0.0001. NCOA4: ****P < 0.0001, ***P = 0.0001, and *P = 0.0244; FTH1: ****P < 0.0001, ***P = 0.0007, and **P = 0.0075. Quadruplicate nMOST measurements, ordinary one-way analysis of variance (ANOVA) with multiple comparisons, α = 0.05. Error bars, SD. (**B**) Immunoblotting of lysates from HeLa control cells treated for 3 days ±U18666A. (**C**) Cells were stained with Filipin and immunostained with α-LAMP1 and α-HA to detect TMEM192^-HA^ in lysosomes, followed by confocal microscopy. Scale bar, 10 μm. (**D**) Cells from (C) examined with 3D-SIM. Scale bar, 2 μm. (**E**) Immunostaining of indicated cells with α-LAMP1, α-LC3B; nuclei stained with 4′,6-diamidino-2-phenylindole (DAPI). LC3B intensities across individual Lamp1-positive lysosomes. Scale bars, 5 and 2 μm (insets). Right: LC3/LAMP1 quantification. (**F**) 3D-SIM reconstructions of HeLa control cells treated for the indicated times with U18666A and *NPC1^−/−^* immunostained for α-LAMP1 and α-panGABARAP. N, position of nucleus. (**G**) Confocal images of control and 4KO cells (fed) immunostained with α-FTH1 and α-HA to detect TMEM192^HA^. Cholesterol-rich lysosomes were stained with Filipin, and nuclei were stained with DNA SPY555. Scale bar, 20 μm. (**H**) Images from (G) were quantified by measuring Mander’s overlap between FTH1 signal and lysosome mask provided by α-HA staining. Data from 12 image stacks per condition; genotype (# cells, fed): Control(3307), *LIPA*^−/−^ (1996), *GAA*^−/−^ (1401), *NPC1*^−/−^ (1211), and *NPC2*^−/−^ (1629). Error bars, SD. (**I**) Confocal images of *NPC2*^−/−^ cells immunostained for α-LAMP1 and α-FTH1, with Filipin-marked lysosomes. A single z-slice is shown. Scale bar, 2 μm. (**J**) 3D-SIM reconstructions of *NPC1*^−/−^ or *NPC2*^−/−^ cells immunostained with α-FTH1 and surface volume of cholesterol-rich lysosomes marked by Filipin.

The patterns of protein alterations confirmed the major findings of the large-scale LSD screen and identified alterations in the abundance of several mitochondrial functional categories, as well as lysosomal and autophagy proteins in *LIPA^−/−^*, *NPC1*^−/−^, and *NPC2*^−/−^ mutants, but not *GAA*^−/−^ cells (fig. S2I). In particular, we confirmed an increase in the abundance of Ub-binding receptors SQSTM1, TAX1BP1, and NBR1, as well as LC3B (MAP1LC3B), under both fed and EBSS-treated conditions, particularly in *NPC1*^−/−^ and *NPC2*^−/−^ cells (Fig. 4A), a result that recapitulates previous studies examining lysosomes in *NPC1*^−/−^ cells (*15*). This phenotype appeared to be a direct reflection of loss of NPC pathway function, as selective inhibition of the NPC1 transporter with the small-molecule U18666A in control HeLa^TMEM192-HA^ cells resulted in analogous accumulation of SQSTM1, LC3B, and GABARAP proteins, as determined by immunoblotting of cell extracts (Fig. 4B).

### Juxta-lysosomal autophagy receptors and cargo in *NPC* mutants

Lysosomes of *NPC1*^−/−^ and *NPC2*^−/−^ HeLa^TMEM192-HA^ cells exhibited a swollen morphology, with Filipin-positive lumen and puncta corresponding to lysosome-associated membrane glycoprotein 1 (LAMP1) and TMEM192^HA^, known to localize in the limiting lysosomal membrane, typically decorating the outer layers of the Filipin-positive core (Fig. 4, C and D, and fig. S3A). Previous work (*15*) concluded that LC3B accumulated within the lysosomal lumen in *NPC1*^−/−^ cells and proposed defects in lysosomal degradation as being responsible for receptor accumulation. To examine this possibility further, we measured the abundance and localization of LC3B in control and *NPC* mutants. Loss of either NPC protein resulted in significantly increased LC3B and SQSTM1 puncta (fig. S3, B and C). However, in *NPC2^−/−^* cells, the majority of LC3B puncta were detected at juxta-lysosomal locations, as opposed to the core region of lysosomes (Fig. 4E). We did not observe significant accumulation of LC3B in control cells, as expected (Fig. 4E and fig. S3D). This ATG8 accumulation phenotype was replicated by treatment of control cells with increasing durations of U18666A treatment, resulting in the accumulation of analogous juxta-lysosomal GABARAP puncta around aberrant swollen lysosomes (Fig. 4F), analogous to observations in *NPC1*^−/−^ cells.

In addition to general autophagy receptors, *NPC1*^−/−^ and *NPC2*^−/−^ cells accumulated NCOA4, a receptor for ferritinophagy (*19*, *20*), and the cargo proteins FTH1 and ferritin light chain (FTL) (Fig. 4A). Ferritin, a cage-like protein complex composed FTH1 and/or FTL proteins, binds ~4500 Fe^+3^ atoms and also promotes the conversion of Fe^+2^ species to Fe^+3^ to reduce reactivity (*24*). FTH1 complexes are delivered to lysosomes as well as late endosomes via NCOA4, which directly binds a conserved motif in FTH1 and promotes encapsulation within an autophagosome (*19*, *25*–*27*). As with general autophagy receptors, NCOA4 and FTH1 accumulated in response to NPC1 inhibition with U18666A (Fig. 4B), indicating a direct effect of NPC pathway inhibition. Consistent with a defect in ferritinophagy, FTH1-positive puncta significantly accumulated in *NPC1^−/−^* and *NPC2^−/−^* (Fig. 4, G and H), and at closer inspection, such puncta were found in the proximity of lysosomes (Fig. 4I). The localization of juxta-lysosomal FHT1 puncta was verified using three-dimensional structured illumination microscopy (3D-SIM), with the majority of FTH1 signal external to or only partially embedded in Filipin-positive structures (Fig. 4J).

Previous work has established a pathway for conjugation of ATG8 proteins to single membranes (CASM) such as lysosomes in response to various signals through an ATG16L1-dependent mechanism (*28*). As such, we examined whether increased juxta-lysosomal ATG8 proteins in *NPC* mutant cells reflected CASM. Although control cells display increased GABARAP-II species when treated with the CASM activator mucolipin 1 synthetic activator 5 (MLSA5) (*29*), an agonist of the lysosomal transient receptor potential channel mucolipin 1 (TRPML1) cation channel (*30*), levels of GABARAP-II were already very high in *NPC1*^−/−^ cells and were not further increased with MLSA5 (fig. S3E). Moreover, levels of GABARAP-II remained elevated in the presence of human vacuolar protein-sorting-associated protein 34 (VPS34) inhibitor, which blocks canonical autophagy (fig. S3E), suggesting that elevated GABARAP-II and LC3B-II levels may not be reversible within the time frame of this experiment. Therefore, as an orthogonal approach to examine a possible role for CASM in ATG8 accumulation, we used SopF, a *Samonella* effector protein known to inhibit CASM by blocking interaction between ATG16L1 and Vacuolar ATPase (adenosine triphosphatase) (*31*, *32*). We blocked NPC1 activity in control cells that were transiently expressing green fluorescent protein (GFP)–SopF using U18666A (24 hours) and examined ATG8 proteins and lysosomal colocalization by imaging in cells with or without GFP-SopF (fig. S3F). SopF expression led to a ~50% reduction in the fraction of LAMP1-positive lysosomes with coincident GABARAP, but this effect was independent of NPC1 inhibition (fig. S3F). Total LC3B puncta and increased lysosomal size verified NPC1 inhibition in this experiment (fig. S3F). Thus, we conclude that autophagy receptors such as ATG8 proteins accumulate in a juxta-lysosomal localization, with no obvious role for CASM under the conditions tested. Together, these data indicate an inability of *NPC1* and *NPC2* mutants to successfully deliver multiple types of autophagic receptors, and FTH1 cargo to the lysosomal lumen is the driving force behind the autophagic deficiency, as opposed to a defect in the process of lysosomal degradation, per se (*15*).

### Defective endocytic cargo delivery to lysosomes in *NPC2*^−/−^ cells

**Fig. 5.**
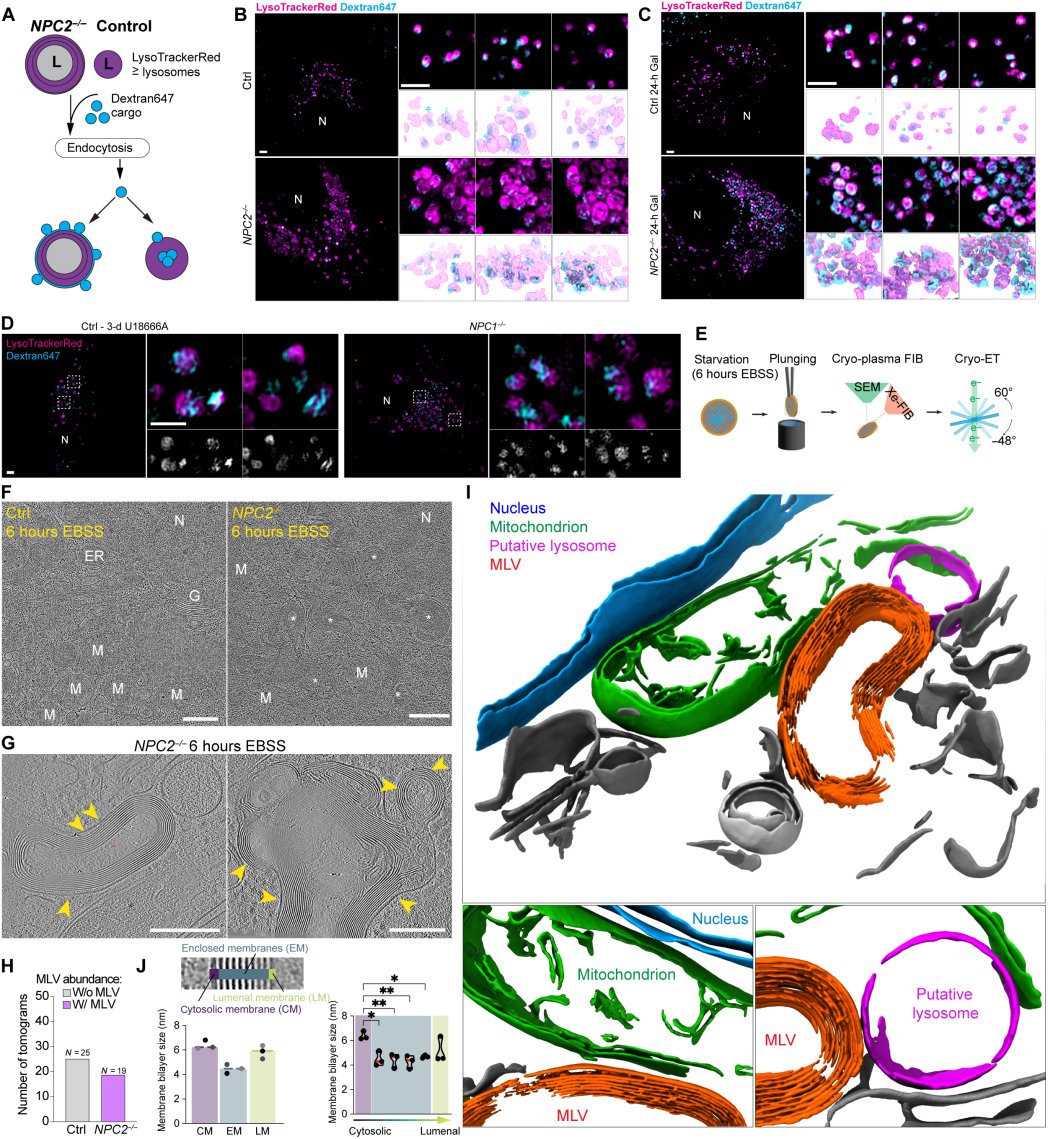
Multilamellar membranes in *NPC2*^−/−^ lysosomes visualized by cryo-ET. (**A**) Schematic showing dextran endocytosis and lysosomal incorporation in control and *NPC2*^−/−^ cells. In control cells, endocytosis successfully delivers dextran to the lysosomal lumen via vesicle fusion. Fusion-dependent delivery of dextran is reduced *NPC2*^−/−^ cells, with successful fusion events resulting in dextran present between the limiting lysosomal membrane and the first internal membrane. (**B**) Indicated cells were treated with dextran conjugated with Alexa647 dye and imaged by live-cell 3D-SIM. 3D-SIM reconstructions are shown below. Scale bars, 2 μm. (**C**) As in (B), but with galactose (Gal) growth media [24 hours (h)]. Scale bars, 2 μm. (**D**) Control cells were treated with the NPC1 inhibitor U18666A (3 days) alongside *NPC1^−/−^* cells with dextran conjugated with Alexa647 dye and imaged by live-cell 3D-SIM. 3D-SIM reconstructions are shown below. Scale bars, 2 μm. (**E**) Cryo-ET workflow. (**F**) Lamella overviews of control and *NPC2*^−/−^ cells with 6 hours of EBSS treatment. Scale bars, 500 nm. (**G**) Tomogram slice of multilamellar vesicles (MLVs) in *NPC2*^−/−^ cells. Scale bars, 200 nm. (**H**) Quantification of MLV-containing tomograms from control and *NPC2*^−/−^ cells. Number of tomograms analyzed is indicated. (**I**) 3D renderings of a segmented *NPC2*^−/−^ tomogram. Zoom-ins highlighting close proximity between MLV (orange) with mitochondria (green), with a putative lysosome (pink) below. (**J**) Quantification of membrane bilayer size (left) and distance between membrane leaflets (right) across three tomograms for the cytosolic membrane (CM), the enclosed membranes (EM), and the luminal membrane (LM). Quantification of spacing between individual membranes: CM to first EM (left), between EMs (middle), and EM to LM (right). *P = 0.011 and 0.21 and **P = 0.0086 and 0.0052. Data based on triplicate lamellae, ordinary one-way ANOVA with multiple comparisons, α = 0.05. Error bars, SD. ER, endoplasmic reticulum; G, Golgi; M, mitochondria; N, Nucleus.

The finding that autophagic receptors and FTH1 cargo accumulate at juxta-lysosomal locations in *NPC1*^−/−^ and *NPC2*^−/−^ cells suggested an inability of lysosomes to efficiently fuse with autophagosomes. Endocytosis represents a distinct pathway for lysosomal trafficking, where late endosomes fuse with lysosomes to facilitate degradation of endocytic cargo. We hypothesized that if lysosomes from *NPC2*^−/−^ cells are defective in this process, then cargo-containing vesicles would accumulate in juxta-lysosomal locations analogous to that observed with autophagy receptors (Fig. 4A). To examine this hypothesis, control and *NPC2*^−/−^ cells under fed conditions were incubated with the extracellular endocytic cargo Dextran647 and LysoTrackerRed (to visualize lysosomes). Live-cell 3D-SIM revealed that while Dextran647 puncta in control cells were largely coincident with the lysosomal lumen, the Dextran647 signal in *NPC2*^−/−^ cells was largely excluded and appeared to form partial “halo”-type structures surrounding lysosomes (Fig. 5B). The simplest explanation for these results is that attempted fusion of Dextran647-loaded endosomes with cholesterol-laden lysosomes places Dextran647 signal coincident with the limiting membrane without allowing full access to the lysosomal lumen. As with LC3B, SQSTM1, and FTH1, much of the Dextran647 signal in either cells grown on glucose or galactose remained juxta-lysosomal (Fig. 5, B and C), consistent with defective fusion. Juxta-lysosomal Dextran647 signal was also observed in control cells treated for 3 days with U18666A to an extent similar to that observed in *NPC1*^−/−^ cells (Fig. 5D), indicating that defects in cholesterol efflux from lysosomes can rapidly lead to defects in lysosomal function independent of prolonged efflux defects in the context of cells constitutively lacking *NPC1*.

As an additional measure of lysosomal function, we examined whether *NPC1*^−/−^ cells maintained the ability to release cations upon TRPML1 activation. We loaded HeLa control and *NPC1^−/−^* cells with the cell-impermeable calcium indicator Oregon Green 488 BAPTA-5N and measured its lysosomal signal with or without MLSA5 treatment (fig. S3G). Control cells displayed a reduction in lysosomal calcium indicator, consistent with cation efflux upon TRPML1 activation. In contrast, lysosomes in *NPC1*^−/−^ cells retained calcium indicator, suggesting that efflux was inhibited in the absence of NPC1 (fig. S3G). These data indicate that multiple aspects of lysosomal function are compromised in cells lacking NPC pathway function.

### Multilamellar vesicles in *NPC2*^−/−^ cells correlate with lyso-PC abundance by nMOST

The observations described above led us to examine lysosomal ultrastructure at higher resolution than is possible by light microscopy. Consistent with a previous study (*33*), electron microscopy (EM) revealed numerous enlarged vesicular structures in *NPC1*^−/−^ and *NPC2*^−/−^ cells, containing densely stained membrane structures which were rare in control cells (fig. S3H). To examine the morphology of these structures in situ without fixation artifacts, we made use of semiautomated cryo–plasma focused ion beam (cryo-PFIB) milling paired with cryo-ET. (Fig. 5E and fig. S4A). Consistent with classical EM, *NPC2^−/−^* cells treated with EBSS harbored numerous multilamellar vesicles (MLVs). These structures were frequently seen in low-magnification EM overviews but rarely in control cells (Fig. 5F, white stars). On the basis of the size and cellular localization of these MLVs, we hypothesized that these structures correspond to the aberrant, cholesterol-filled lysosomes characterized by fluorescent microscopy (Fig. 5, C, D, and F, and fig. S2B). Analysis of the corresponding tomograms (25 for control, 19 for *NPC2^−/−^* cells) revealed that the majority of MLVs in *NPC2*^−/−^ cells were structurally aberrant, containing as many as 17 highly organized membranes surrounding a lumen (Fig. 5, F to H). Segmentation of tomograms highlights the highly organized membrane structures with *NPC2*^−/−^ MLVs (Fig. 5I), as well as their close proximity to mitochondria. The intermembrane distance inside MLVs was highly regular (2.6 ± 0.2 nm between membranes at half maximum), whereas the distance of the outer limiting membrane to the first enclosed membrane was highly variable across MLVs (Fig. 5G, yellow arrowheads, and fig. S4, B to D). For the limiting membrane, both leaflets were clearly distinguishable with full width at half maximum (FWHM) of 6.4 ± 0.4 nm (Fig. 5J). However, the interleaflet space of the enclosed membranes and the luminal membrane could not be resolved at the magnification used and showed FWHM of 4.5 ± 0.4 and 5.8 ± 0.5 nm, respectively. (Fig. 5J and fig. S4, B to D).

Accumulation of MLVs suggested potential alterations in lipid abundance, possibly as a consequence of defective cholesterol efflux. We therefore used nMOST data to globally examine lipid alterations in *NPC* mutant cells with or without starvation, identifying alterations in two major lipid classes. First, CE abundance was generally elevated in whole-cell lipidomics of *NPC2^−/−^* cells, as indicated by a skew of CE species toward the upper end of ranked abundance relative to control cells (figs. S2H and S4E). This was particularly evident for CE species with chain lengths shorter than 20 carbons (fig. S4E). Retrieval of lipid-protein cross-ome correlation networks of select high-abundance CE species in *NPC2^−/−^* cells from the LSD-nMOST dataset revealed correlation with autophagy and ferritinophagy markers (fig. S4F). Second, we observed strong accumulation of lyso-PC species, major building blocks of cellular membranes, but not Lyso-PE species and other types of phospholipids, in *NPC2*^−/−^ cells under both fed and starvation conditions (fig. S4G). Lyso-PC species likewise carry autophagy/ferritinophagy signatures (fig. S4H). Third, increased lyso-PC appeared selective for *NPC* mutants, as lyso-PC levels in *LIPA^−/−^* or *GAA^−/−^* cells were less strongly affected (fig. S4I). Lyso-PC species that were enriched in *NPC2^−/−^* cells had, on average, chain lengths of <20 carbons (fig. S4J). We speculate that characteristic and tightly packed spacing of MLVs in *NPC2*^−/−^ cells (Fig. 5I) could reflect the generation of multilamellar membranes enriched in shorter-chain lyso-PC species.

### OXPHOS complex and cristae defects in *NPC2*^−/−^ cells

**Fig. 6.**
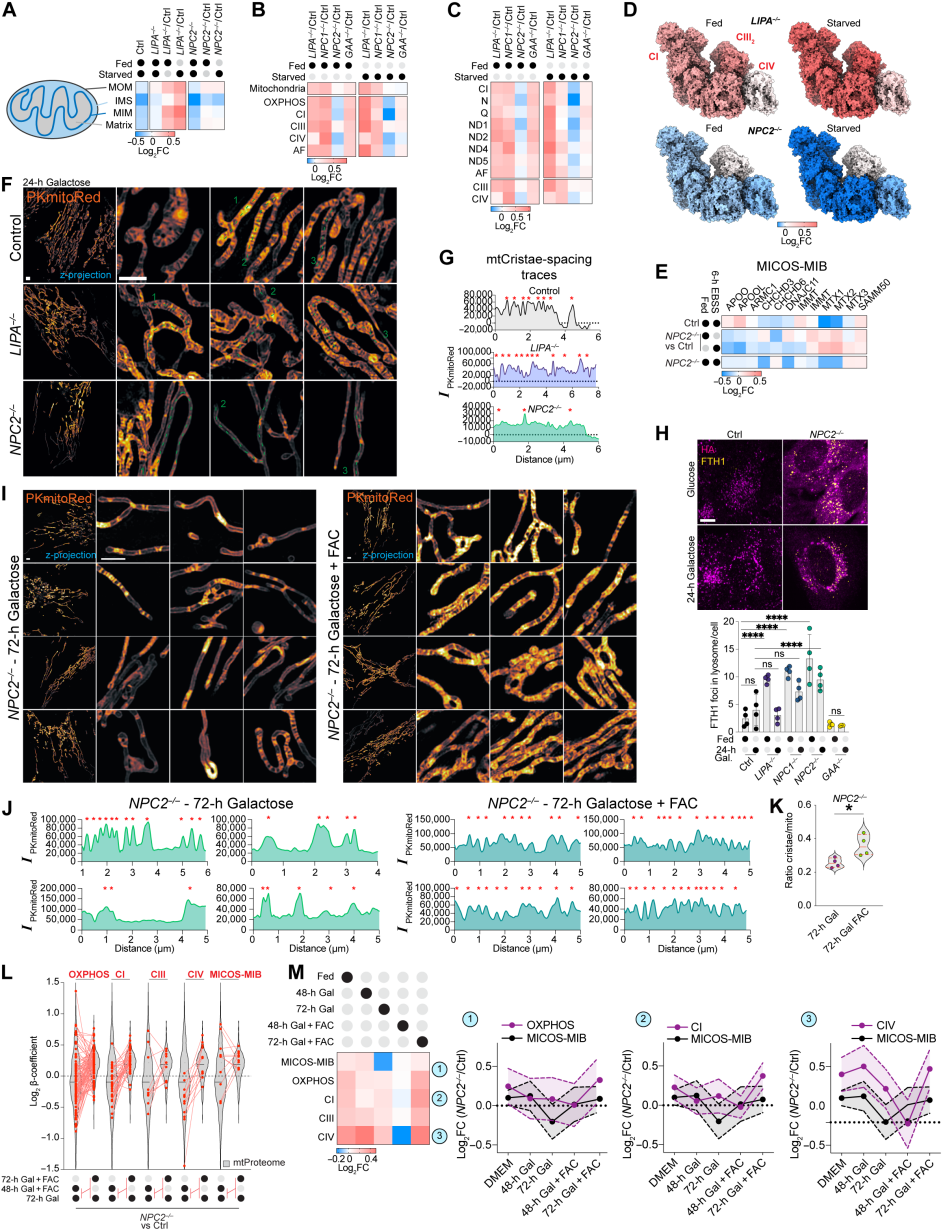
Mitochondrial cristae/OXPHOS system defects in *NPC2*^−/−^ cells and amelioration by extracellular iron. (**A**) Mitochondrial compartment heatmap (log_2_FC) in fed or EBSS-treated control and 4KO cells (*n* = 4). (**B**) Log_2_FC values for OXPHOS modules for 4KO cells in fed or EBSS-treated cells (*n* = 4). (**C**) Log_2_FC of replisome submodule abundance [quadruplicate fed versus EBSS treated control or 4KO cells (nMOST data)]. (**D**) Abundance from (B) mapped onto CI, CIII_2_, and CIV structure [Protein Data Bank (PDB): 5XTH]. Color panel, log_2_FC values. (**E**) MICOS-MIB complex heatmap for 4KO cells (normalized with control). Biological quadruplicate nMOST measurements. (**F**) Z-projections of live-cell 3D-SIM images from control, *LIPA^−/−^*, and *NPC2^−/−^* cells (galactose, 24 hours) stained with IMS dye PKmitoRed. Scale bar, 2 μm. (**G**) Line plots of dashed lines from (F). (**H**) Control and *NPC2*^−/−^ cells grown in glucose (fed) or galactose (24 hours) followed by immunostaining with α-HA to detect TMEM192^HA^ and α-FTH1 to detect Ferritin. Scale bar, 10 μm. Right: Quantification of FTH1 signal overlapping with lysosome staining/cell. Data from biological quadruplicates per sample (each replicate containing five to nine stacks); ****P < 0.0001, ordinary two-way ANOVA, multiple comparisons, α = 0.05. Error bars, SD. (**I**) Z-projections of live-cell 3D-SIM images from control and *NPC2*^−/−^ cells after galactose (72 hours) ± FAC and stained with the IMS dye PKmitoRed. Scale bars, 2 μm. (**J**) Line plots of individual mitochondria from (I). Red asterisks, positions of cristae. (**K**) Violin plot depicting the ratio of cristae to mitochondria ± FAC. Data based on 132 (72-hour galactose) or 148 (72-hour galactose + FAC) segmented planes of ROI stacks from data in (I); *P = 0.0242, unpaired *t* test. (**L**) Log_2_ β-coefficient for OXPHOS subunits and individual subcomplexes. (**M**) Log_2_FC (*NPC2*^−/−^/control) for indicated protein complexes under the indicated conditions (time in galactose ± FAC addback) (*n* = 3). ns, not significant.

The finding that *NPC1^−/−^* and *NPC2^−/−^* cells exhibited accumulation of FTH1 and NCOA4 led us to explore whether iron-dependent processes downstream of lysosomal function and ferritinophagy are affected. We analyzed nMOST data from *NPC1*^−/−^, *NPC2*^−/−^, *LIPA*^−/−^, *GAA*^−/−^, and control cells in either fed or EBSS-treated (6 hours) states for alterations in two systems that are heavily reliant on iron availability—cytosolic Fe-S cluster assembly machinery (*34*) and components of the mitochondrial OXPHOS system, which contain several subunits with Fe-S clusters (*35*). We were particularly interested in the OXPHOS system as GO terms related to this were found enriched in *NPC2*^−/−^ cells by nMOST (figs. S1J and S2I) (*36*). The inner-membrane space (IMS) compartment in *NPC2^−/−^* cells was especially depleted of organelle-annotated proteins (Fig. 6A and table S4). When normalized to control, we observed a reduction in a cohort of complex I (CI) subunits in *NPC2*^−/−^ cells under starvation conditions, which was most pronounced for components of the N-module [log_2_ fold change (FC) ~ −0.41] (Fig. 6, A to D, and fig. S5, A and B). Five of the eight subunits within the N-module of CI contain Fe-S clusters, consistent with a reliance on iron for stability and/or assembly (*35*, *37*). The abundance of the Q-module, which contains four subunits with Fe-S clusters, as well as CIV, was also slightly reduced in *NPC2*^−/−^ mutants in the presence of EBSS (log_2_FC ~ −0.1) (Fig. 6, A to D, and fig. S5, A and B). These alterations were in contrast with the abundance of mitochondrial Fe-S cluster assembly machinery in *NPC1*^−/−^ and *NPC2*^−/−^ cells, which was largely unchanged or slightly increased when compared with control cells (fig. S5, C and D).

The intimate connectivity between OXPHOS complex assembly and mitochondrial cristae structure (*38*), together with the finding that several MICOS-MIB complex components are reduced in *NPC2*^−/−^ cells relative to control cells (particularly in EBSS conditions; Fig. 6E), led us to examine mitochondrial ultrastructure. Using a photo-stable and cell-permeable IMS dye PKmitoRed, combined with live-cell 3D-SIM, we observed alterations in cristae morphology particularly in *NPC2*^−/−^ cells in the presence of galactose to enforce OXPHOS utilization (Fig. 6, F and G). Unlike control cells, which displayed regularly spaced cristae, *NPC2*^−/−^ cells displayed an unexpected morphology reflective of altered cristae structure, including extensive regions of mitochondria, often near the cell periphery, that lacked obvious cristae as indicated by line plots (Fig. 6, F and G; red stars indicate cristae bridge). In contrast with *NPC2*^−/−^ cells, *LIPA*^−/−^ cells displayed an increased number of cristae, albeit with less regular intervals (Fig. 6, F and G), in line with the overall increase in OXPHOS and mitochondrial proteome compared to control cells (Fig. 6, B and C, and fig. S5B). These data indicate multiple defects in mitochondrial morphology and proteome abundance in the absence of *NPC2*.

### Alleviation of cristae morphology defects in *NPC2*^−/−^ cells by extracellular iron

The apparent defect in ferritinophagy in *NPC2*^−/−^ cells led us to examine whether mitochondrial defects could be mechanistically linked with iron availability. As an alternative to iron mobilization by ferritinophagy, we tested whether extracellular iron delivered to the cytoplasm via transferrin-dependent endocytosis could rescue cristae morphology. Transferrin-associated iron undergoes endocytosis, where the reduced pH of the endosome allows iron release from transferrin and transport to the cytosol via the DMT1/SLC11A2 proton-coupled metal ion transporter (*39*).

We first verified that FTH1 abundance, juxta-lysosomal FTH1 localization, and endolysosomal system fusion phenotypes are retained in *NPC2*^−/−^ cells grown in galactose (Fig. 6H). We then examined cristae morphology in *NPC2*^−/−^ cells grown in galactose in the presence or absence of FAC (ferric ammonium citrate; 72 hours) as an extracellular iron source. Both the frequency of cristae and their spacing were significantly rescued by FAC addition (Fig. 6, I to K). Change of growth medium slightly reduced the average lysosomal size and percentage of Filipin-positive lysosomes, but FAC addition did not further influence these parameters (fig. S5, E and F), indicating that FAC does not rescue lysosomal defects associated with *NPC* deficiency. We examined mitochondrial membrane potential (ΔΨm) in *NPC2*^−/−^ and control cells by determining the ratio of tetramethylrhodamine methyl ester (TMRE) to MitoTrackerDeepRed (as mask). Control cells grown on galactose (72 hours) displayed high membrane potential, as indicated by the prominent TMRE signal (fig. S5H). In contrast, *NPC2*^−/−^ cells displayed reduced ΔΨm, a phenotype which was partially ameliorated with the addition of FAC (fig. S5H). Consistent with the partial rescue of cristae morphology and ΔΨm after FAC addback, *NPC2*^−/−^ cell growth under galactose media conditions was increased by ~28% (fig. S5I). Together, these data indicate that these aspects of mitochondrial dysfunction in *NPC2*^−/−^ cells are partially alleviated by providing a ferritinophagy-independent route for iron delivery to the cytosol and mitochondria.

### MICOS-MIB complex proteome remodeling in *NPC2*^−/−^ cells by extracellular iron

To examine the effect of FAC addition on the mitochondrial proteome in an unbiased manner, we performed two 18-plex tandem mass tagging (TMT) proteomic experiments examining total proteomes from control or *NPC2^−/−^* cells under five conditions: Dulbecco’s modified Eagle’s medium (DMEM), 48 hours galactose ± FAC, 72 hours galactose ± FAC (fig. S5J and table S5). Mirroring the observations from the 4KO-nMOST dataset, we observed an overall increase in the abundance of autophagy receptors in *NPC2*^−/−^ (including LC3B, SQSTM1, TAX1BP1, NCOA4, FTH1, and FTL); however, other proteins related to iron homeostasis and FeS cluster assembly were largely unaltered (fig. S5, K to M).

We next examined the mitochondrial proteome. *NPC2*^−/−^ cells cultured for 72 hours in galactose with FAC displayed a slight increase in the mitochondrial proteome compared to galactose conditions alone; however, focusing on proteins associated with mitochondrial OXPHOS complexes revealed differential changes in response to galactose and FAC addback (fig. S5, N and O). Mapping of protein group abundance on mitochondrial subcompartments highlighted a spatiotemporal element in mitochondrial proteome remodeling during growth media switch and iron addback (fig. S5P). *NPC2*^−/−^ cells grown in galactose (72 hours) displayed a reduction in the abundance of MICOS-MIB complex components when compared with control cells (fig. S5, Q and R), in accordance with the observed reduction in cristae (Fig. 6, F, G, and I). In contrast, the abundance of MICOS-MIB complex subunits was largely rescued by FAC for either 48 or 72 hours, consistent with imaging data described above (fig. S5, Q and R). Alterations in the abundance of individual MICOS subunits are displayed schematically in fig. S5S. Together, these results indicate that imbalances in iron homeostasis can lead to reversible changes in mitochondrial ultrastructure.

### OXPHOS complex proteome remodeling in *NPC2*^−/−^ cells by extracellular iron

We next systematically examined the effect of FAC on electron transport chain components in *NPC2*^−/−^ cells. The average abundance of OXPHOS subunits as a cohort increases over the FAC addback time course (fig. S5O). Focusing on CI, changes in FeS cluster–containing N- and Q-modules revealed the largest abundance shifts in the presence of FAC. At early time points, these modules appear destabilized, followed by stabilization of the membrane arm modules (ND1,2,4,5) and a subsequent increase in N-module proteins between 48 and 72 hours of FAC addback (fig. S5T). Distinct patterns were observed for individual respirasome (CI-CIII_2_-CIV) modules and assembly factors, as shown for *NPC2*^−/−^ versus control cells (fig. S6, A and B). A linear increase in CI abundance was observed upon FAC addback when comparing [*NPC2*^−/−^/Control], which was coincident with increased levels of respective assembly factors. However, CIV displayed a biphasic pattern with an initial decrease in abundance at the 48-hour time point (fig. S6, A and B).

Next, we evaluated the effect that the growth media conditions and FAC addback have on the proteome in the context of *NPC2*^−/−^ versus control (β-coefficient; Fig. 6L and fig. S6C). Log_2_ β-coefficients for the transition from 48 to 72 hours in the presence of FAC and galactose were not significant for the proteome at large (β-coefficient = 0.008); however, although individual subunits displayed differential alterations in abundance, generally detectable increases in mitochondria (β-coefficient = 0.16) and especially OXPHOS components (β-coefficient = 0.21) were observed. This included all but one subunit of N- and Q-modules, as well as all the nuclear genome–encoded CIV subunits (fig. S6C). Consistent with the differences observed on the abundance between 48 and 72 hours, β-coefficients across OXPHOS components at these time points revealed differential changes that were not see with MICOS-MIB subunits (Fig. 6L). Recovery of CI and CIV subunits primarily occurred during the 48- to 72-hour interval and was preceded temporally by MICOS-MIB rescue (Fig. 6L). We also examined alterations in the abundance of proteins known to function in assembly of Fe-S clusters in either the cytoplasm or the mitochondria (fig. S5M). The log_2_FC *NPC2*^−/−^/control values of most cytosolic Fe-S cluster assembly components (*40*) were slightly decreased in response to 72 hours of FAC, while, in contrast, the abundance of mitochondrial Fe-S cluster assembly proteins was either increased or remained constant, with the exception of ABCB7, which functions to transport [2Fe-2S]-(glutathione)_4_ from the mitochondria to the cytosol (*40*). These data are consistent with an elevated Fe-S cluster biogenesis pathway in mitochondria of *NPC2*^−/−^ cells treated with FAC when compared with control cells. The effect of FAC addback on the abundance of OXPHOS components is summarized schematically in fig. S6D.

### Effect of FAC on OXPHOS abundance during in vitro neurogenesis

**Fig. 7.**
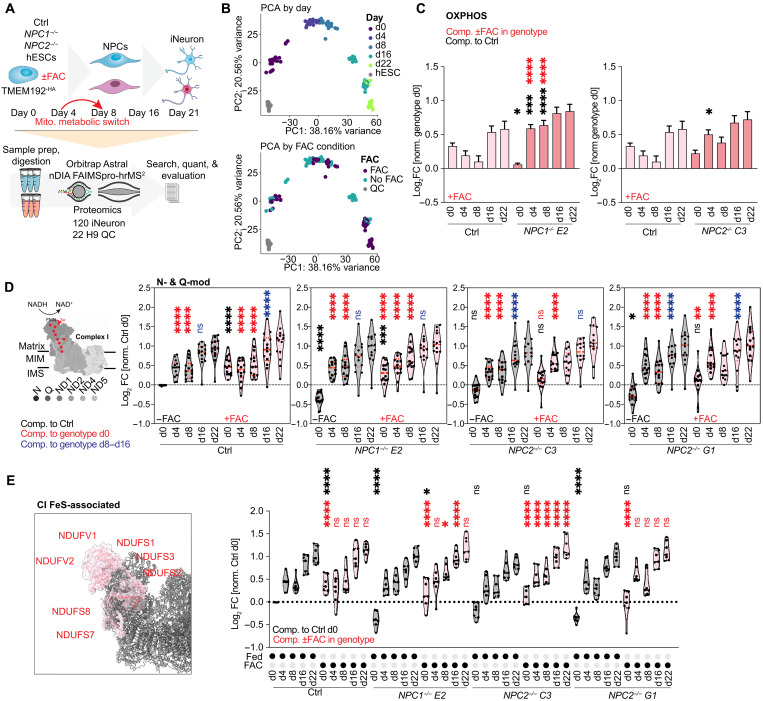
Proteomic analysis of *NPC* mutants during neurogenesis with iron addback. (**A**) Proteomic analysis of NGN2-driven neurogenesis ± iron (*n* = 3 per time point). (**B**) PCA of LFQ data, color coding, day of differentiation, or ±FAC. (**C**) Mean log_2_FC (normalized within genotype day 0) mitochondrial OXPHOS components with FAC. Black asterisks, comparison to control cells; red asterisks, comparison ± FAC within genotype. *N* = 3; *NPC1^−/−^* clone E2 d0: *P = 0.0286; d4: ***P = 0.0003; d8, d4 + FAC, and d8 + FAC: ****P < 0.0001. *NPC2^−/−^* clone C3 d0: *P = 0.0420. Ordinary two-way ANOVA, multiple comparisons, α = 0.05. Error bars, SEM. (**D**) Violin plots of log_2_FC (normalized to day 0 control) N- and Q-module of CI. Left: CI and FeS clusters (based on PDB: 5XTH). Abundance comparisons during time course ± FAC treatment are shown for each genotype (gray, −FAC; red, +FAC). Black asterisks, comparison to control cells; red asterisks, comparison within genotype. Blue asterisks, comparison of days 8 to 16 within genotype. *N* = 3; control d0, d4, and d8: ****P < 0.0001; d4 and d8 + FAC: ****P < 0.0001; d8 to d16: ****P < 0.0001. *NPC1^−/−^* clone E2 d0: ****P < 0.0001; d0 + FAC: ***P = 0.0002; all other: ****P < 0.0001. *NPC2^−/−^* clone C3: ****P < 0.0001. *NPC2^−/−^* G1 d0: *P = 0.0396; d0 + FAC: **P = 0.0041; all other: ****P < 0.0001. Ordinary two-way ANOVA, multiple comparisons, α = 0.05. Error bars, SEM. (**E**) FeS-associated CI N- and Q-modules (red) overlayed with rest of the complex in gray (PDB: 5XTH). Violin plots of log_2_FC (normalized to control, day 0) FeS-associated proteins. Abundance during differentiation time course ± FAC treatment (gray for −FAC, red for +FAC). Black asterisks, statistical comparison to control cells; red asterisks, comparison within genotype ± FAC. *N* = 3; control: ****P < 0.0001. *NPC1^−/−^* clone E2 d0: *P = 0.0489; d8 + FAC: *P = 0.0146; all other: ****P < 0.0001. *NPC2^−/−^* clone C3: ****P < 0.0001. *NPC2^−/−^* G1: ****P < 0.0001. Ordinary two-way ANOVA, multiple comparisons, α = 0.05. Error bars, SEM.

Mouse models for *NPC* deficiency displayed disturbances in iron homeostasis (*41*), potentially affecting iron availability in brain tissue. We set out to investigate whether the ferritinophagy defect observed in HeLa cells would likewise affect mitochondrial OXPHOS complexes in neuronal models. Stem cells undergo a metabolic switch from glycolysis to OXPHOS during early stages of neuronal differentiation in vitro (*42*). We therefore asked whether *NPC* deficiency and/or FAC addback in this system would affect electron transport chain abundance during NGN2-driven in vitro neurogenesis. Control, *NPC1*^−/−^, and *NPC2*^−/−^ stem cells containing an inducible AAVS1-NGN2 cassette (see Materials and Methods) were differentiated over a 3-week period with or without FAC and subjected to proteomic and phenotypic analysis (Fig. 7A). As expected, *NPC* mutants displayed an increase in the number of LAMP1-positive lysosomes at day 14 of differentiation (fig. S7A), consistent with results in HeLa cells. For proteomic analysis, cells from triplicate cultures at five time points during differentiation were subjected to label-free narrow-window data-independent acquisition (nDIA) proteomics (Fig. 7A and table S6) (*43*). Despite the number and complexity of the samples, the number of proteins quantified remained stable across runs, and randomly inserted stem cell QC lysates (*n* = 22) demonstrated high reproducibility, with samples distinguished by PCA (Fig. 7B and fig. S7, B and C). Neither loss of *NPC1^−/−^* or *NPC2^−/−^*, confirmed by proteomics (fig. S7D), nor FAC addition altered the known timing of alterations in pluripotency and differentiation markers (fig. S7E) (*42*), and as expected, FAC addition promoted the accumulation of FTH1 by immunoblotting and proteomics (fig. S7F and S7G).

Analysis of the abundance of OXPHOS components during the differentiation time course revealed three core findings: First, in stem cells, the global abundance of OXPHOS components was reduced in *NPC1^−/−^* and *NPC2^−/−^* mutants, when normalized with control cells, and this was rescued in the presence of FAC (fig. S7H). Thus, stem cells recapitulate the central finding in HeLa cells. Second, during differentiation, total OXPHOS abundance increases across all genotypes, consistent with conversion from glycolysis to oxidative phosphorylation, but FAC had a more profound effect on *NPC1^−/−^* and *NPC2^−/−^* mutants especially on day 4 or day 8 than in control cells (Fig. 7C and fig. S7I). Third, the effect of FAC addition on the abundance of N- and Q-modules as a whole, as well as iron-sulfur cluster containing components within these modules, was also evident in stem cells (Fig. 7, D and E). Together, these data indicate that features of the effect on *NPC1^−/−^* or *NPC2^−/−^* deficiency and iron regulation are present in the stem cell in vitro neurogenesis paradigm.

## DISCUSSION

Here, we report the nMOST workflow for simultaneous analysis of lipids and proteins from the same sample and its application to a collection of more than two dozen cell lines lacking individual LSD genes. Cross-correlation analysis between lipids and proteins across various genotypes reveals numerous molecular fingerprints associated with specific LSD alleles, providing a resource for further mechanistic discovery. We performed accompanying proteomic experiments using data-dependent acquisition (DDA)–TMTpro and nDIA-LFQ to validate and follow up the biological insights we gained from nMOST, and to our knowledge, this work represents one of the most in-depth attempts in profiling LSDs, spanning multiple cell types and growth conditions. This resource can also be combined with cell type–specific lysosomal proteome analysis (*8*) to further understand the relationship between lysosomal protein abundance and specific LSD loss of function alleles.

We observed a prominent and selective phenotype with NPC1, NPC2, and TPP1 mutants involving accumulation of autophagy regulators, which correlated with accumulation of lyso-PC. Through 3D-SIM imaging, we provided evidence for a block in autophagic clearance wherein autophagic receptors (e.g., LC3B) or cargo (e.g., FTH1) accumulate in juxta-lysosomal locations with evidence of defective delivery of cargo to the lysosomal lumen. This phenotype in *NPC2*^−/−^ cells correlated with the formation of multilamellar lysosomes, which we speculate may be reflective of the increased abundance of lyso-PC in these cells (Fig. 2C). Previous studies (*16*, *44*) have implicated decreased lysosomal cleavage of cargo as well as defects in autophagosome-lysosome fusion, but the underlying mechanisms were unclear. We suggest that multilamellar membranes within lysosomes observed by cryo-ET reduce the ability of lysosomes to efficiently fuse with either autophagosomes or endosomes, thereby limiting delivery of cargo to the lysosomal lumen.

Among the autophagic cargo that accumulated in *NPC1*^−/−^ and *NPC2*^−/−^ cells was the ferritin cage protein FTH1, which was juxta-lysosomal based on super-resolution imaging. Given that a block to ferritin degradation in the lysosome would be expected to reduce iron availability, we examined complexes known to rely on Fe-S clusters for their production, leading to the identification of mitochondrial electron transport chain complexes as being reduced in cells lacking *NPC2* (fig. S6D, left). Loss of OXPHOS complexes correlated with reduced cristae number and MICOS-MIB complexes in *NPC2*^−/−^ cells (fig. S6D, left). Delivery of iron to cells through endocytosis results in initial accumulation of MICOS-MIB subunits at 48 hours (fig. S6D, middle), which supports further assembly of OXPHOS complexes at 72 hours, with near-full restoration of OXPHOS complexes and cristae number (fig. S6D, right). The behavior of MICOS-MIB and OXPHOS complexes and the effects on cristae number are consistent with the self-reinforcing role that these components play in formation and stabilization of cristae (*38*). We note that while cells lacking *NPC1* also accumulate FTH1, the corresponding phenotypes and particularly the mitochondrial alterations seen in *NPC2*^−/−^ cells appear more pronounced, despite only 5% of *NPC* patients carry mutations in the *NPC2* gene (*45*). This phenotype held also true in stem cell–derived iNeurons. Previous studies have described an imbalance of iron metabolism and hematological abnormalities in *NPC1* mouse models and in patients with Niemann-Pick disease type C1 (*41*), although to our knowledge, these alterations have not be directly linked to disruption of OXPHOS. Further studies are required to understand the extent to which an inability to promote iron mobilization by autophagy and concomitant effects on mitochondrial function are linked with defects observed in patients. We note that while disruption of iron homeostasis in budding yeast has been linked with mitochondrial defects, the underlying mechanisms appear to be distinct (*46*). The discovery platform we have described here and its application to relevant cell lineages linked with LSDs may facilitate identification of molecular defects or pathways with relevance to disease.

## MATERIALS AND METHODS

All details and catalog numbers can be found in the key resource table (table S8). Protocols can be found on protocols.io (dx.doi.org/10.17504/protocols.io.5qpvokmmzl4o/v1).

### Cell culture

HeLa TMEM192-3xHA cells (referred to as HeLa^TMEM192-HA^) (*21*) were maintained in DMEM, supplemented with 10% (vol/vol) fetal bovine serum (FBS), 5% (vol/vol) penicillin-streptomycin (P/S), 5% (vol/vol) GlutaMAX, and 5% (vol/vol) nonessential amino acids (NEAA) at 37°C, 5% O_2_. Unless otherwise noted, we refer to independently grown and handled cultures as biological replicates to distinguish from assays performed on identical samples (i.e., technical replicates). For galactose growth conditions, galactose-containing DMEM was prepared from glucose-free DMEM supplemented with 10% (vol/vol) dialyzed FBS, 25 mM d-galactose, 5% (vol/vol) P/S, 5% (vol/vol) GlutaMAX, 5% (vol/vol) sodium pyruvate and uridine (50 μg/ml).

### Gene editing

Generation of LSD mutants in the HeLa^TMEM192-HA^ or H9 AAVS-NGN2^TMEM192-HA^ background (*21*, *47*) was facilitated using CRISPR-Cas9 with target sites determined using CHOPCHOP (https://chopchop.cbu.uib.no/). Guide RNAs were ligated into the px459 plasmid (Addgene plasmid #62988), and cells were transfected using Lipofectaime LTX reagent (Thermo Fisher Scientific, 15338100), according to the manufacturer’s instructions. Two days posttransfection, single, puromycin-resistant cells were sorted into 96-well dishes containing 300 μl of full growth medium. To generate *NPC1^−/−^* or *NPC2^−/−^* in H9 embryonic stem cells (ESCs), 0.6 μg of single guide RNA (sgRNA) was incubated with 3 μg of SpCas9 protein for 10 min at room temperature and electroporated into 2 × 10^5^ wild-type (WT) H9 cells using the Neon Transfection System (Thermo Fisher Scientific) and sorted into 96-well dishes containing 300 μl of full growth medium (composition as described above). Single cells were allowed to grow into colonies and duplicated for multiplex sequencing. Genomic DNA samples were obtained by incubating cells in 30 μl of PBND [50 mM KCl, 10 mM tris-HCl (pH 8.3), 2.5 mM MgCl_2_-6H_2_O, 0.45% NP-40, and 0.45% Tween 20] with protease K (40 μg/ml) at 37°C for 5 min and heated to 55° and 95°C for 30 and 15 min, respectively. The first round of polymerase chain reaction (PCR) was performed to amplify the target region using gene-specific primers that contain partial Illumina adaptor sequences (i.e., forward primer: 5′-ACACTCTTTCCCTACACGACGCTCTTCCGATCT[n]_18–22_ -3′; reverse primer: 5′-GTGACTGGAGTTCAGACGTGTGCTCTTCCGATCT[n]_18–22_ -3′, [n]_18–22_ represent gene-specific sequences). The resulting PCR products with adapter-modified ends can be further amplified in the second round of PCR by universal primers containing attachment sites for the flow cell and index sequences (i.e., forward primer: 5′-AATGATACGGCGACCACCGAGATCTACACTCTTTCCCTACACGACGCTCTTCCGATC-3′; reverse primer: 5′-CAAGCAGAAGACGGCATACGAGAT-[n]_8_-GTGACTGGAGTTCAGACGTGTGCT-3′, [n]_8_ represents index sequences). The final PCR products were purified using a QIAquick PCR purification kit (Qiagen, 28106). Sequencing was performed using Miseq Reagent kits v2 on Illumina Miseq following the denature and dilute libraries guide of the Miseq system, and sequencing data were analyzed by Outknocker program (www.OutKnocker.org). Knockout candidates were confirmed by Western blot on whole-cell lysates or by proteomics. The sgRNAs were generated using the GeneArt Precision gRNA Synthesis Kit (Thermo Fisher Scientific) according to the manufacturer’s instruction and purified using the RNeasy Mini Kit (Qiagen). The sgRNA target sequences and sequencing results can be found in table S1. HeLa^TMEM192-HA^ control, *GRN*^−/−^ and *HEXA*^−/−^, as well as the H9 AAVS-NGN2^TMEM192-HA^ control cells have been previously reported (*9*, *21*, *47*).

### iNeuron differentiation

Human ES cells (H9, WiCell Institute) were cultured in E8 medium (*42*, *48*) on Geltrex-coated tissue culture plates with daily medium change. Cells were passaged every 4 to 5 days with 0.5 mM EDTA in 1× Dulbecco’s phosphate-buffered saline (Thermo Fisher Scientific). SpCas9 and AsCas12a/AsCpf1 expression plasmids pET-NLS-Cas9-6xHis (Addgene plasmid #62934) and modified pDEST-his-AsCpf1-EC, generated by deleting the maltose binding protein (MBP) sequence from plasmid pDEST-hisMBP-AsCpf1-EC (Addgene plasmid #79007), were transformed into *Rosetta*(*DE3*)*pLysS*-competent cells (Novagen), respectively, for expression. SpCas9 and AsCas12a/AsCpf1 proteins were purified as described elsewhere (*49*, *50*). Briefly, cells expressing SpCas9 (0.5 mM isopropylthio-β-galactoside, 14-hour induction) were lysed in FastBreak buffer (Promega Inc.), and the NaCl concentration was adjusted to 500 mM. Extracts were centrifuged at 38,000*g* for 10 min at 4°C, and the supernatant was incubated with nickel-nitrilotriacetic acid (Ni-NTA) resin for 1 hour. The resin was washed extensively with 50 mM tris (pH 8.0), 500 mM NaCl, 10% glycerol, 20 mM imidazole, and 2 mM tris(2-carboxyethyl)phosphine (TCEP) before elution with this buffer supplemented with 400 mM imidazole. Proteins were diluted two volumes/volume in phosphate-buffered saline (PBS) and fractionated on a Heparin-Sepharose column using a 0.1 to 1.0 M NaCl gradient. Cas9-containing fractions were stored in PBS, 20% glycerol, and 2 mM TCEP at −80°C. AsCpf1 expression was induced similarly, and cells were pelleted by centrifugation. Cells were lysed by sonication in 50 mM Hepes (pH 7), 200 mM NaCl, 5 mM MgCl_2_, 1 mM dithiothreitol (DTT), and 10 mM imidazole supplemented with lysozyme (1 mg/ml) and protease inhibitors (Roche complete, EDTA-free). After centrifugation (16,000*g* for 30 min), the supernatant was incubated with Ni-NTA resin, the resin was washed with 2 M NaCl, and bound proteins were eluted with 250 mM imidazole and buffer-exchanged into lysis buffer lacking MgCl_2_ and imidazole before storage at −80°C.

For human ES cell conversion to iNeurons, cells were expanded and plated at 2 × 10^4^/cm^2^ on Geltrex-coated tissue plates in DMEM/F12 supplemented with 1× N2, 1× NEAA (Thermo Fisher Scientific), human brain-derived neurotrophic factor (BDNF; 10 ng/ml; PeproTech), human neurotrophin-3 (NT-3; 10 ng/ml; PeproTech), mouse laminin (0.2 μg/ml; Cultrex), Y-27632 (10 μM; PeproTech), and Doxycycline (2 μg/ml; Alfa Aesar) on day 0. On day 1, Y-27632 was withdrawn. On day 2, medium was replaced with neurobasal medium supplemented with 1× B27 and 1× GlutaMAX (Thermo Fisher Scientific) containing BDNF, NT-3, and doxycycline (1 μg/ml). Starting on day 4, half of the medium was replaced every other day thereafter. On day 7, the cells were treated with Accutase (Thermo Fisher Scientific) and plated at 3 to 4 × 10^4^/cm^2^ on Geltrex-coated tissue plates. Doxycycline was withdrawn on day 10. Following day 12 of differentiation, 50% ND2 media was changed every other day. FAC was added if necessary.

### NPC1 inhibitor treatments

Indicated cells were differentiated (if necessary), seeded in six-well plates, and treated with the NPC1 inhibitor U18666A for 1 to 3 days at the indicated concentrations in regular growth medium at 37°C. Before harvesting, cells were washed twice with 1× PBS and harvested for further experimental procedures.

### MLSA5 and VPS34 inhibitor treatments

Indicated cells were differentiated (if necessary) and seeded in six-well plates. Cells were treated for 2 or 3 hours with the VPS34 inhibitor SAR405 (1 μM) or 2 hours with MLSA5 (10 μM) in regular growth medium at 37°C. A double treated sample was also included. Before harvesting, cells were washed twice with 1× PBS and harvested for further experimental procedures.

### Western blotting

At the indicated times, ES cells, iNeurons, or HeLa cells were washed on ice in 1× PBS and harvested, and the pellet was washed with 1× PBS and resuspended in 8 M urea buffer [8 M urea, 150 mM tris (pH 7.4), 50 mM NaCl, and PhosSTOP phosphatase inhibitor cocktail]. Resuspended cell lysates were sonicated for 10 s, and the debris was pelleted at 13,000 rpm for 10 min. Protein concentration was determined by bicinchoninic acid (BCA) assay according to the manufacturer’s instructions (Thermo Fisher Scientific, 23227). Indicated amounts of proteins were resuspended in 1× LDS + 100 mM DTT and boiled for 10 min at 85°C. Equal amounts of protein were loaded, run on 4 to 20% bis-tris and 8% tris NuPAGE gels for 5 min at 100 V and 5 min at 150 V, and then run at 200 V for the required time. Gels were transferred via a wet transfer system onto polyvinylidene difluoride membranes for Western Blotting. Chemiluminescence and colorimetric images were acquired using a Bio-Rad ChemiDoc MP imaging system. Images from Western blots were exported and analyzed using Image Lab and ImageJ/Fiji (*51*).

### Proteomics

#### Sample preparation for nMOST

For samples used for technical evaluation of MOST, the bead-enabled, accelerated, monophasic multiomic method was used (*13*). Silica-coated superparamagnetic beads (700 nm; SeraSil-Mag) were washed and resuspended in water for a concentration of 75 μg/μl, while frozen cell pellets were being thawed on ice. Two hundred microliters of acetonitrile (ACN), 600 μl of *n*-butanol, and 200 μl of beads containing water were added to samples. After vortex, samples were sonicated for 5 min at 14°C. Beads were immobilized by magnet, and 100 μl of the supernatant was aliquoted, dried down, and reconstituted in 300 μl *n*-butanol:isoproponal (IPA):water (8:23:69, v/v/v) in an amber autosampler vial for lipids (*52*, *53*). The remaining supernatant was removed. The beads were reconstituted in Rapid Digestion Buffer (Promega) diluted to 75% by water with 2 mM TCEP and 40 mM 2-chloroacetamide (CAA). After incubation for 10 min at room temperature, trypsin (Promega) was added in a 20:1 ratio (protein-to-trypsin). The samples were incubated in a thermomixer for 40 min at 60°C and 1000 rpm. Formic acid (FA) was added to terminate digestion. Peptides were desalted by a Sep-Pak (Waters) C18 column, dried down in SpeedVac (Thermo Fisher Scientific), and reconstituted in 0.2% FA.

For HeLa whole-cell extracts or lyso-immunoprecipitation (IP) samples [generated as described (*9*, *21*); dx.doi.org/10.17504/protocols.io.ewov14pjyvr2/v2], 300-μl mixture of *n*-butanol:ACN:water (3:1:1, v/v/v) was added. Samples were bath sonicated for 5 min at 14°C. After centrifugation at 14,000*g* for 5 min, 50 μl of the lipid-containing supernatant was transferred to autosampler vials with glass insert, dried down in SpeedVac, and resuspended in 50 μl of *n*-butanol:IPA:water (8:23:69, v/v/v) (*52*, *53*). The remaining samples were maintained at −80°C until protein digestion. For protein digestion of whole-cell extracts, the samples were thawed on ice and centrifuged at 14,000g for 5 min. The remaining supernatant was removed from samples. One hundred microliters of lysis buffer [8 M urea, 100 mM tris (pH 8.0), 10 mM TCEP, and 40 mM CAA] was added. The samples were bath sonicated for 5 min at 14°C and vortexed for 15 min. Protein concentration was determined by Thermo protein BCA assay (reducing agent compatible). Lysyl endopeptidase (LysC) (FUJIFILM Wako) was added to samples in a 50:1 ratio (protein-to-LysC) and incubated on a rocker for 4 hours at room temperature. The urea was diluted to 2 M by 300 μl of 100 mM tris (pH 8.0). Trypsin was added to samples in a 50:1 ratio (protein-to-trypsin) and incubated on a rocker overnight at room temperature. For protein digestion of lyso-IP samples, 60 μl of 6 M GnHCl and 100 mM tris were added to the sample to solubilize proteins from being aggregated on beads. The samples were bath sonicated 5 min at 14°C, incubated in a thermomixer for 5 min at 100°C and 600 rpm, and then incubated for 2 hours at 80°C and 600 rpm. Beads were immobilized by magnet, and the supernatant was transferred to a 96-well plate. GnHCl was diluted to 2 M by adding 120 μl of 100 mM tris, 10 mM TCEP, and 40 mM CAA. LysC was added to samples in a 50:1 ratio (protein-to-LysC) and incubated on a rocker for 4 hours at room temperature. GnHCl was diluted to 0.4 M by adding 420 μl of 100 mM tris (pH 8.0). Trypsin was added to samples in a 50:1 ratio (protein-to-trypsin) and incubated on a rocker overnight at room temperature. Trifluoroacetic acid (10%) was added to terminate digestion. After centrifugation at 12,000*g* for 5 min, digested peptides were desalted by StrataX 10-mg 96-well plate (Phenomenex), dried down in SpeedVac, and reconstituted in 0.2% FA. The peptide concentration was determined by Thermo peptide BCA assay.

#### nMOST LC-MS

Separation was performed on an in-house packed bridged ethylene hybrid (BEH) C18 capillary column (28 cm in length by 75 μm in inner diameter by 1.7 μm in particle size) at 60°C and an Ultimate3000 system (Thermo Fisher Scientific). Column packing was described previously (*54*). Mobile phase A consisted of 0.2% FA in water. Mobile phase B consisted of 0.2% FA and 5 mM ammonium formate in IPA/ACN (90:10, v/v). Lipids were loaded onto column first and then peptides at 0% mobile phase B. Mobile phase B increased to 70% over 80 min for scanning tandem mass spectrometry (MS/MS) spectra of peptides and increased to 100% over 26 min for scanning MS/MS spectra of lipids. The column was washed at 100% mobile phase B for 3 min and reequilibrated at 0% mobile phase B for 10 min. Eluting analytes were analyzed by an Orbitrap Eclipse Tribrid mass spectrometer (Thermo Fisher Scientific). Spray voltage was 2 kV. Ion transfer tube temperature was 275°C. MS^1^ scan range was 200 to 1600 mass/charge ratio (*m/z*). MS^1^ resolution was 240,000 (at 200 *m/z*). Source radio frequency (RF) was 35. MS^1^ automatic gain control (AGC) target was 300%. MS^1^ injection time was 50 ms. Duty cycle was 1 s. Polarity was positive. For proteomic data acquisition from 0 to 80 min, precursor selection range was 300 to 1350 *m/z*. Charge states were 2 to 5. Dynamic exclusion was 10 s. Isolation width was 0.5 *m/z*. Precursors were fragmented by higher-energy collisional dissociation (HCD) with a normalized collision energy (NCE) of 25%. MS^2^ mass spectra were acquired in data-dependent mode using ion trap turbo speed. MS^2^ scan range was 150 to 1350 *m/z*. MS^2^ AGC target was 300%. MS^2^ injection time was 14 ms. For lipidomic data acquisition from 80 to 120 min, precursor selection range was 300 to 1600 *m/z*. Charge states were 1 to 2. Dynamic exclusion was 10 s. Isolation width was 0.7 *m/z*. Precursors were fragmented by HCD with a stepped NCE of 27 ± 5%. MS^2^ mass spectra were acquired in data-dependent mode using ion trap rapid speed. MS^2^ scan range was auto. MS^2^ AGC target was 300%. MS^2^ injection time was 17 ms. Real-time library search and complementary collision-induced dissociation were used for glycerophospholipids and sphingomyelins as described previously (*14*). For large-scale LSD samples, to improve the throughput of the analysis, the total LC time was set down to 105 min. Peptides were eluted and were analyzed from 0 to 70 min, while lipids were eluted and were analyzed from 70 to 105 min.

#### nMOST MS data process

For proteomics, raw data files were processed by MaxQuant (version 2.0.3.0). The database was canonical plus isoforms downloaded from UniProt in December 2021. The match between runs was on. MS/MS spectra were not required for LFQ comparisons. For lipidomics, raw data files were processed using Compound Discoverer 3.1 (Thermo Fisher Scientific) and LipiDex (*55*). Peak detection required a signal-to-noise ratio (SNR) of 1.5, a minimum peak intensity of 5 × 10^5^, and a maximum peak width of 0.75 min. The chromatographic peaks were grouped into compound groups by a retention time tolerance of 0.5 min and a mass tolerance of 10 parts per million (ppm). Peaks were removed if the peak areas of sample over blank were <3-fold. An in silico–generated lipid spectral library (LipiDex_HCD_Formic) was used for MS/MS spectra searching. The threshold of dot product score was 500, and the threshold of reverse dot product score was 700. MS^2^ spectra were annotated at the molecular species level if the minimum spectral purity was at least 75%; otherwise, sum compositions were reported. The lipid identification was further filtered for adducts, dimers, in-source fragments, misidentified isotopes, and mismatched retention time by LipiDex and the degreaser module of LipiDex 2 (https://github.com/coongroup/LipiDex-2) (*56*). Cross-ome correlation analysis between lipids and proteins was analyzed via nMOST. Proteins and lipids were correlated using the Kendall rank correlation approach [R function corr(); the resulting matrix was filtered for lipids or proteins with at least two correlations |>0.4| Tau]. The filtered matrix was further clustered using hierarchical clustering and subset into 18 protein clusters and 14 lipid clusters (*k*-means). Members of each cluster were evaluated for enrichment in GO terms (cellular components) or lipid class using a Fisher’s exact test.

#### Neurogenesis with iron supplementation and label-free nDIA proteomics

Control, *NPC1^−/−^*, and *NPC2^−/−^* were seeded on Geltrex-coated tissue culture plates and differentiated according to the methods stated above. For day 0 (d0) and d4 time points, cells were seeded in triplicates in coated 12-well plates. For all other time points, cells were replated at day 4 in triplicates into coated 12-well plates. FAC was added during regular media changes. Cells were dissociated using 0.5 mM EDTA-PBS, washed in PBS, and pelleted at 2000*g* for 5 min, the supernatant was aspirated, and the pellet was snap-frozen and stored at −80°C.

Cell pellets were lysed in 140 μl of 8 M urea and 100 mM tris (pH 8.0) by a syringe with a 21G needle. Protein concentration was measured by protein BCA assay. A total of 10 mM TCEP and 40 mM chloroacetamide were added for reduction and alkylation. LysC (FUJIFILM Wako) was added to samples in a 50:1 ratio (protein-to-LysC), and the samples were incubated on a rocker for 4 hours at room temperature. The urea was diluted to 2 M with 100 mM tris (pH 8.0). Trypsin was added to samples in a 50:1 ratio (protein-to-trypsin), and the samples were incubated on a rocker overnight at room temperature. Digested peptides were desalted using Waters 25-mg Sep-Pak tC18 96-well plates (186002319). The concentration of desalted peptides was measured by peptide BCA assay.

Separation was performed on a C18 capillary column (30 cm in length by 100 μm in inner diameter) packed with Accucore C18 resin (2.6 μm, 150 Å; Thermo Fisher Scientific) at 55°C and an EASY-nLC 1200 system (Thermo Fisher Scientific). The flow rate was set to 450 nl/min. Mobile phase A consisted of 0.125% FA in ACN/water (5:95, v/v). Mobile phase B consisted of 0.125% FA in ACN/water (95:5, v/v). When peptides were loaded on the column, mobile phase B increased from 5 to 23% over 22 min, increased to 100% over 2 min, and stayed at 100% for 6 min. Eluting peptides were analyzed by an Orbitrap Astral mass spectrometer (Thermo Fisher Scientific). Spray voltage was 2.2 kV. Ion transfer tube temperature was 290°C. The field asymmetric ion mobility spectrometry (FAIMS) compensation voltage (CV) was −55 V. MS^1^ scan range was 380 to 980 *m/z*. MS^1^ resolution was 240,000 (at 200 *m/z*). Source RF was 50%. MS^1^ AGC target was 500%. MS^1^ injection time was 50 ms. Precursors were isolated in the quadrupole by an isolation width of 2 *m/z* and fragmented by HCD with an NCE of 27%. MS^2^ mass spectra were acquired in data-independent mode in the Astral analyzer. MS^2^ scan range was 110 to 2000 *m/z*. MS^2^ AGC target was 500%. MS^2^ max injection time was 3 ms.

#### Astral LFQ nDIA MS data process

Raw files were converted to mzML format using MSConvert (*57*) and analyzed using FragPipe (v22.0) with DIA-NN (v1.8.2) (*58*–*60*). All settings were kept default.

#### TMTpro 18plex proteomics

##### Proteomic sample preparation

Sample preparation of proteomic analysis of whole-cell extract from HeLa control and mutant lysates was performed according to a previously published study (*42*). Replicate cell cultures were grown and treated independently and are considered biological replicates in the context of TMT experiments. Cells were washed twice with 1× PBS and harvested on ice using a cell scraper in 1× PBS. Cells were pelleted via centrifugation for 5 min (5000*g*, 4°C) and washed with 1× PBS before resuspending in lysis buffer [urea, 150 mM tris (pH 7.4), 150 mM NaCl, and protease and phosphatase inhibitors added]. After a 10-s sonication, and optional French pressing through a G25 needle, lysed cells were pelleted, and protein concentration of the clarified sample was determined using a BCA kit (Thermo Fisher Scientific, 23227). One hundred micrograms of protein extract of each sample were incubated for 30 min at 37°C with 5 mM TCEP for disulfide bond reduction with subsequent alkylation with 25 mM chloroacetamide for 10 min at room temperature with gentle shaking. Methanol-chloroform precipitation of samples was performed as follows: To each sample, four parts of methanol (MeOH) were added and vortexed, one part of chloroform was added and vortexed, and lastly, three parts of water were added. After vortexing, suspension was centrifuged for 2 min at 14,000*g*, and the aqueous phase around the protein precipitate was removed using a loading tip. Peptides were washed twice with MeOH, resuspended in 200 mM 3-[4-(2-Hydroxyethyl)piperazin-1-yl]propane-1-sulfonic acid (EPPS) (pH 8), and digested for 2 hours with LysC (1:100) at 37°C, followed by trypsin digestion (1:100) at 37°C overnight with gentle shaking.

##### TMT labeling

Fifty microliters of digested samples was labeled by adding 10 μl of TMT reagent (stock: 20 mg/ml in ACN) together with 10 μl of ACN [final ACN concentration of approximately 30% (v/v)] for 2 hours at room temperature before quenching the reaction with hydroxylamine to a final concentration of 0.5% (v/v) for 15 min. The TMTpro-labeled samples were pooled together at a 1:1 ratio, resulting in a consistent peptide amount across all channels. Pooled samples were vacuum centrifuged for 1 hour at room temperature to remove ACN, followed by reconstitution in 1% FA, and samples were desalted using C18 solid-phase extraction (200 mg; Sep-Pak, Waters) and vacuum centrifuged until near dryness.

##### Basic pH reverse-phase high-performance LC

Dried peptides were resuspended in 10 mM NH_4_HCO_3_ (pH 8.0) and fractionated using basic pH reverse-phase high-performance LC (*61*). Samples were offline fractionated into 96 fractions over a 90-min run by using an Agilent LC1260 with an Agilent 300 Extend C18 column (3.5-μm particles, 2.1 mm in inner diameter, and 250 mm in length) with mobile phase A containing 5% ACN and 10 mM NH_4_HCO_3_ in LC-MS–grade H_2_O and mobile phase B containing 90% ACN and 10 mMNH_4_HCO_3_ in LC-MS–grade H_2_O (both pH 8.0). The 96 resulting fractions were then pooled in a noncontinuous manner into 24 fractions (*62*). This set of 24 fractions was divided into 2 × 12 sets (even or odd numbers), acidified by addition of 1% FA and vacuum centrifuged until near dryness. One set (12 samples) was desalted via StageTip, dried, and reconstituted in 10 μl of 5% ACN and 5% FA before LC-MS/MS processing.

##### Mass spectrometry acquisition

For HeLa whole-cell proteomics, data collection was performed on a Orbitrap Fusion Lumos Tribrid mass spectrometer (Thermo Fisher Scientific, San Jose, CA), coupled with a FAIMS Pro device and a Proxeon EASY-nLC1200 LC (Thermo Fisher Scientific). A total of 10% of resuspended samples were loaded on a 35-cm analytical column (100 mm in inner diameter) packed in-house with Accurcore150 resin (150 Å, 2.6 mm; Thermo Fisher Scientific, San Jose, CA) for LC-MS analysis. Peptide separation was performed with a gradient of ACN (0.1% FA) from 3 to 13% (0 to 83 min) and 13 to 28% (83 to 90 min) during a 90-min run. LC-MS/MS was combined with three optimized CV parameters on the FAIMS Pro Interface to reduce precursor ion interference (*63*). DDA was performed by selecting the most abundant precursors from each CV’s (−40/−60/−80) MS1 scans for MS/MS over a 1.25-s duty cycle. The parameters for MS1 scans in the Orbitrap include a 400 to 1600 *m/z* mass range at 60,000 resolution [at 200 Thomson (Th)] with 4 × 10^5^ AGC (100%) and a maximum injection time (IT) of 50 ms. Most abundant precursors (with 120-s dynamic exclusion ± 10 ppm) were selected from MS1 scans, isolated using the quadrupole (0.6 Th isolation), fragmented with HCD (36% NCE), and subjected to MS/MS (MS2) in the Orbitrap detector at 50,000 resolution, 5× AGC, 110 to 200 *m/z* mass range, 86 ms IT, and with 120-s dynamic exclusion ± 10 ppm.

##### Data processing

Raw mass spectra were converted to mzXML, and monoisotopic peaks were reassigned using Monocle (*64*) and searched using Comet (*65*) against all canonical isoforms found in the Human reference proteome database (UniProt Swiss-Prot 2019-01; https://ftp.uniprot.org/pub/databases/uniprot/previous_major_releases/release-2019_01/) as well as against sequences from commonly found contaminant proteins and reverse sequences of proteins as decoys, for target-decoy competition (*66*). For searches, a 50-ppm precursor ion tolerance and 0.03-Da product ion tolerance for ion trap MS/MS as well as trypsin endopeptidase specificity on C terminus with two maximum missed cleavages were set. Static modifications were set for carbamidomethylation of cysteine residues (+57.021 Da) and TMTpro labels on lysine residues and N termini of peptides (+304.207 Da); variable modification was set for oxidization of methionine residues (+15.995 Da). Peptide-spectral matches were filtered at 2% false discovery rate (FDR) using linear discriminant analysis (picked FDR method, based on XCorr, DeltaCn, missed cleavages, peptide length, precursor mass accuracy, fraction of matched product ions, charge state, and number of modifications per peptide), additionally restricting PSM Xcorr > 1 and peptide length > 6 and after a 2% protein FDR target filtering (*67*). PSM reporter ion intensities were quantified. Quantification was performed using a 0.003-Da window around the theoretical TMT reporter *m/z* and filtered on precursor isolation specificity of >0.5 in the MS1 isolation window, and the output was filtered using summed SNR across all TMT channels > 100. MS statsTMT (*68*) was performed on peptides with >200 summed SNR across TMT channels. For each protein, the filtered peptide–spectrum match TMTpro raw intensities were summed and log_2_-normalized to create protein quantification values (weighted average) and normalized to total TMT channel intensity across all quantified PSMs (adjusted to median total TMT intensity for the TMT channels) (*69*). Log_2_-normalized summed protein reporter intensities were compared using a Student’s *t* test, and *P* values were corrected for multiple hypotheses using the Benjamini-Hochberg adjustment (*70*). Linear model analysis was performed as described (*71*). Subcellular and functional annotations were based on a previously published list of high-confidence annotations (*72*) [“high” and “very high” confidence, additional manual entries from (*42*, *47*); the AmiGO Pathway online tool and mitochondrial annotation were based on MitoCarta 3.0 (*73*)]. A part of heatmaps was created using Morpheus (https://software.broadinstitute.org/morpheus). GO enrichment was performed with ShinyGO (*74*).

### Microscopy

#### *Live-cell spinning disk microscopy*—*General acquisition parameters*

For analysis of organelles using live-cell spinning disk microscopy, cells were seeded into either 24-well 1.5 high-performance glass-bottom plates (Cellvis, P24-1.5H-N) or μ-Slide eight-well, glass-bottom plates (ibidi, #80807) and further cultured in the vessel until reaching appropriate confluency for microscopy. Before microscopy, cells were washed in 1× PBS and imaged in FluoroBrite DMEM media. Cells were imaged using a Yokogawa CSU-X1 spinning disk confocal on a Nikon Eclipse Ti2-E motorized microscope. The system is equipped with a Tokai Hit stage top incubator, and imaging was performed at 37°C, 5% CO_2_, and 95% humidity under a Nikon Plan Apo 60×/1.40 numerical aperture (NA) immersion oil objective lens. Fluorophores were excited in a sequential manner with a Nikon LUN-F XL solid-state laser combiner (laser line–laser power): 405 nm–80 mW, 488 nm–80 mW, 561 nm–65 mW, and 640 nm–60 mW using a Semrock Di01-T405/488/568/647 dichroic mirror. Fluorescence emissions were collected through a Chroma ET455/50m (405 nm), Chroma ET525/36m (488 nm), Chroma ET 605/52m (561 nm), and a Chroma ET700/75m (for 640 nm) filters, respectively (Chroma Technologies). Images were acquired with a Hamamatsu ORCA-Fusion BT complementary metal-oxide semiconductor (CMOS) camera (6.5-μm^2^ photodiode, 16-bit) and NIS-Elements image acquisition software. Consistent laser intensity and exposure time were applied to all the samples, and brightness and contrast were adjusted equally by applying the same minimum and maximum display values in ImageJ/Fiji (*51*). Image quantification was performed in ImageJ/Fiji using custom-written batch-macros.

#### Live-cell microscopy for mitochondrial membrane potential measurements

For measuring mitochondrial membrane potential in live cells, HeLa^TMEM192-HA^ control and mutant cell lines were seeded in μ-Slide eight-well chambers and treated according to the experimental plan. Before imaging, cells were incubated with TMRE (1:5000) and MitoTrackerDeepRed (1:10,000) for 1 hour at 37°C and washed twice with PBS, and growth media was replaced before imaging. A total of 5% laser power and 100-ms (568-nm) or 50-ms (640-nm) exposure time was used to image 6-μm z-stacks of cells. Mitochondrial masks were created on the basis of the MitoTrackerDeepRed signal, and TMRE intensities were measured within these masks for evaluation.

#### Measurement of lysosomal pH using live-cell spinning disk microscopy

Day before measurements, 100,000 cells were seeded in a 24-well glass-bottom plate (Cellvis). On the day of measurement, cells were loaded with SiR-Lysosome (1:1000, Cytoskeleton Inc.) and pHLys Red (1:1000, Dojindo) for 1 hour in DMEM + 10% FBS. Stains was then washed out and chased with phenol red-free DMEM + 10% FBS for 3 hours before imaging. For BafA1 treatment, 1 μM BafA1 was treated 2.5 hours into the chase for 30 min before live-cell imaging on a confocal microscope with 20× objective. To establish the pH calibration curve, WT cells were bathed in calibration buffers with pH adjusted to 3, 4, 5, 6, and 7, supplemented with 10 μM monensin. For both experimental and pH calibration conditions, five to six field of views were imaged and analyzed in its entirety. This process was repeated for each independent experiment. Image analysis was performed using Fiji (ImageJ). For each field of view, background subtraction was processed using the rolling ball background subtraction method for each channel. Subsequently, the Otsu’s method was used to threshold the SiR-Lysosome signal to select a region of interest (ROI) corresponding to lysosomes. The selected ROI was applied to the pHLys Red channel and then measured fluorescence intensity. The fluorescence intensity of the pHLys Red channel was then fitted to the calibration curve to calculate the pH value.

#### Measurement of FAC addback on lysosomes and mitochondria in iNeuron using spinning disk microscopy

For measurement of lysosomal morphology ± FAC in iNeurons, human embryonic stem cells (hESCs) were seeded on day 4 of differentiation in μ-Slide eight-well, glass-bottom plates (ibidi, #80807) and further differentiated in the vessel for the indicated times until reaching appropriate confluency and imaged. Iron was supplemented every second day. iNeurons were imaged using a spinning disk confocal microscope (see details above) using a Nikon Plan Apo 60×/1.40 NA immersion oil objective lens. A total of 5% laser power and 100-ms (568-nm) exposure time was used to image 6-μm z-stacks of cells. Image quantification was performed in CellProfiler. LysoTracker signal was segmented using global Otsu thresholding, and the object shape/number was measured. Statistical analysis and plotting of microscopy data were performed in Prism.

#### Measurement of MLSA5 long-term treatment on lysosomes in iNeuron using spinning disk microscopy

For measurement of lysosomal number after long-term MLSA5 treatment in iNeurons, hESCs were seeded on day 4 of differentiation in μ-Slide eight-well glass-bottom plates (ibidi, #80807) and further differentiated in the vessel for the indicated times until reaching appropriate confluency and imaged. A total of 10 μM MLSA5 was supplemented every other day. Cells were treated with 10 μM Oregon Green 488 BAPTA-1, AM, for 16 hours (overnight) as well as incubated for 1 hour at 37°C with LysoTrackerRed (1:10,000). Cells were carefully washed once, and fresh growth media was added to the wells. iNeurons were imaged using a spinning disk confocal microscope (see details above) using a Nikon Plan Apo 60×/1.40 NA immersion oil objective lens. Two hundred–millisecond exposure time at 50% laser power (488 nm), 100-ms exposure time at 10% laser power (568 nm), and 1-min time lapses with 60 time points were used to image the cells. Image quantification was performed in CellProfiler. Statistical analysis and plotting of microscopy data were performed in Prism.

#### Ca^2+^ release from lysosomes in HeLa cells using spinning disk microscopy

For measurement of lysosomal Ca^2+^ content after acute CASM initiation using MLSA5, HeLa control and *NPC1^−/−^* were seeded into μ-Slide eight-well glass-bottom plates (ibidi, #80807). After reaching appropriate confluency, cells were treated with 10 μM Oregon Green 488 BAPTA-5N for 16 hours (overnight) as well as incubated for 1 hour at 37°C with LysoTrackerRed (1:10,000). Cells were incubated for 1 hour with 10 μM MLSA5 before being carefully washed twice with PBS, and fresh growth media was added to the wells. HeLa cells were imaged using a spinning disk confocal microscope (see details above) using a Nikon Plan Apo 60×/1.40 NA immersion oil objective lens. Two hundred–millisecond exposure time at 20% laser power (488 nm) and 200-ms exposure time at 10% laser power (568 nm) were used to image 2-μm z-stacks of cells. Image quantification was performed in CellProfiler. Statistical analysis and plotting of microscopy data were performed in Prism.

#### Immunocytochemical analysis

HeLa cells were fixed with warm 4% paraformaldehyde (Electron Microscopy Science, #15710, purified, EM grade) in PBS at 37°C for 30 min and permeabilized with 0.5% Triton X-100 in PBS for 15 min at room temperature. After three washes with 0.02% Tween 20 in PBS (PBST), cells were blocked for 10 min in 3% BSA–1× PBS at room temperature and washed again three times in PBST. Cells were incubated for 3 hours in primary antibodies in 3% BSA–1× PBS and washed three times with PBST. Secondary antibodies (Thermo Fisher Scientific, 1:400 in 3% BSA–1× PBS) were applied for 1 hour at room temperature. To stain nuclei, Hoechst33342 (1:10,000) was added for 5 min to cells in PBST and lastly washed three times. Filipin staining was performed after fixation for 2 hours at room temperature in PBS (0.05 mg/ml). Primary and secondary antibodies used in this study can be found in the key resource table.

#### *Fixed-cell microscopy*—*General acquisition parameters*

Immunofluorescently labeled HeLa or iNeurons (antibodies are indicated in figures and figure legends, and details are in the key resource table) were imaged at room temperature using a Yokogawa CSU-W1 spinning disk confocal on a Nikon Eclipse Ti2-E motorized microscope equipped with a Nikon Plan Apochromat 40×/0.40 NA air objective lens, Nikon Plan Apochromat 60×/1.42 NA oil objective lens, and a Plan Apochromat 100×/1.45 NA oil objective lens. Signals of 405/488/568/647 fluorophores were excited in a sequential manner with a Nikon LUN-F XL solid-state laser combiner (laser line–laser power): 405 nm–80 mW, 488 nm–80 mW, 561 nm–65 mW, and 640 nm–60 mW using a Semrock Di01-T405/488/568/647 dichroic mirror. Fluorescence emissions were collected with Chroma ET455/50m (405 nm), 488 Chroma ET525/50m (488 nm), 568 Chroma ET605/52m (561 nm), and 633 Chroma ET705/72m (640 nm) filters, respectively (Chroma Technologies). Confocal images were acquired with a Hamamatsu ORCA-Fusion BT CMOS camera (6.5-μm^2^ photodiode, 16-bit) and NIS-Elements image acquisition software. Consistent laser intensity and exposure time were applied to all the samples, and brightness and contrast were adjusted equally by applying the same minimum and maximum display values in ImageJ/Fiji (*51*).

#### Evaluation of ferritin accumulation in lysosomes

The quantitative measurement of FTH1 accumulation inside the lysosomal mask was performed by seeding HeLa^TMEM192-HA^ control and mutant cell lines into 24-well 1.5 high-performance glass-bottom plates (Cellvis, 24-1.5H-N) and treated according to the experimental plan. Fed and treated cells were fixed according to the procedure stated above, stained for ferritin (FTH1), lysosomes (HA), and DNA (SPY-DNA555), and imaged using a Nikon Plan Apochromat 40×/0.40 NA air objective lens. Twelve randomly selected positions (8-μm z-stacks) were acquired using the high content analysis (HCA) module in NIS-Elements. For image analysis, CellProfiler (*75*) was used for the quantitative analysis of FTH1 colocalization with the HA-derived lysosomal mask. Plotting of microscopy data was performed in Prism. Primary and secondary antibodies used in this study can be found in the key resource table.

#### *Effect of GFP-SopF expression* (*CASM*) *on ATGlyation using spinning disk microscopy*

HeLa^TMEM192-HA^ cells were seeded into 24-well 1.5 high-performance glass-bottom plates (Cellvis, 24-1.5H-N, 20,000 per well). The next day, GFP-SopF was transfected using FuGene HD transfection reagent (Promega) according to the manufacturer’s protocol, and a full media exchange was performed the day after. Two days after the transfection, cells were treated for 24 hours with 2 μM U18666A to block NPC1 function. Cells were fixed using ice-cold MeOH (10 min, for LC3B) or paraformaldehyde (30 min, for panGABARAP) and immunofluorescently labeled for GFP, Lamp1, and LC3B/panGABARAP. Images were acquired using a Yokogawa CSU-W1 spinning disk confocal on a Nikon Eclipse Ti2-E motorized microscope using a Plan Apochromat 60×/1.42 NA oil objective lens. Images were analyzed using CellProfiler. Statistical analysis and plotting of microscopy data were performed in Prism. Primary and secondary antibodies used in this study can be found in the key resource table.

### 3D structured illumination microscopy

#### Fixed cell 3D-SIM sample preparations

Fixed cell 3D-SIM samples were prepared as described (*76*). Briefly, HeLa^TMEM192-HA^ control and mutant cell lines were seeded on 18 mm–by–18 mm Marienfeld Precision cover glasses thickness no. 1.5H (tolerance ± 5 μm) and cultured at the indicated conditions/treatments. To mimic NPC1 loss, control cells were treated with 2 μM U18666A for the indicated time points in growth medium. Cells were fixed at 37°C in 4% paraformaldehyde (Electron Microscopy Science) for 30 min and permeabilized for 15 min with 0.5% Triton X-100 in PBS at room temperature. After three washes with 0.02% PBST, cells were blocked for 10 min in 3% BSA–1× PBS at room temperature and washed again three times in PBST. If required, cholesterol molecules were labeled with Filipin for 2 hours at room temperature in 1× PBS (0.1 mg/ml), before washing the sample with PBST twice to remove the excess label. Primary antibody incubation was performed overnight at 4°C with gentle rocking in 3% BSA–1× PBS, followed by three 5-min washes with PBST. Secondary antibody incubation (1:400 in 3% BSA–1× PBS) was performed at room temperature for 1 hour with gentle rocking. Samples were washed three times for 5 min in 1× PBST. Before mounting on glass slides, coverslips were washed once in 1× PBS and mounted in VECTASHIELD (Vector Laboratories, H-1000-10). Primary and secondary antibodies used in this study can be found in the key resource table.

#### *3D-SIM microscopy*—*Acquisition parameters*

3D-SIM microscopy was performed on a DeltaVision OMX v4 using an Olympus 60×/1.42 Plan Apo oil objective (Olympus, Japan). The instrument is equipped with 405-, 445-, 488-, 514-, 568-, and 642-nm laser lines (all ≥ 100 mW), and images were recorded on a front-illuminated scientific Complementary Metal–Oxide–Semiconductor (sCMOS) (PCO Photonics, USA) in 95 MHz, 512 × 512 pixel image size mode, 1× binning, 125-nm z-stepping, and with 15 raw images taken per *z* plane (five phase shifts, three angles). Raw image data were computationally reconstructed using Compute Unified Device Architecture (CUDA)-accelerated 3D-SIM reconstruction code (https://github.com/scopetools/cudasirecon). Optimal optical transfer function was determined via an in-house build software, developed by T. Lambert (Center for Imaging Technology and Education, Harvard Medical School) (GitHub: https://github.com/tlambert03/otfsearch, all channels were registered to the 528-nm output channel, Wiener filter: 0.002, background: 90). ChimeraX was used for 3D renderings when imaging data.

#### Live-cell 3D-SIM sample preparations

MatTek 35-mm dish, high-precision 1.5 coverslips were coated for 2 hours with poly-l-lysine at 37°C before washing excess solution off with three 1× PBS washes. Cells were seeded in dishes and cultured/treated as indicated. On the day of the experiment, cells were incubated with PKmitoRed (1:1000) for 1 hour at 37°C and washed with warm medium to remove excess dye. For assessing fusion competency of lysosomes in NPC2^−/−^ mutants, cells were seeded in MatTek 35-mm dishes (see above) and loaded with Alex647-conjugated Dextran o/n (1:200 dilution) at 37°C. The next day, cells were stained with LysoTrackerRed DND-99 for 1 hour at 37°C (1:5000) and washed twice with PBS, and the medium was replaced with a fresh, warm growth medium. If necessary, 2 μM U18666A was added to control cells for the indicated times to block NPC1 function.

#### *Live-cell 3D-SIM microscopy*—*Acquisition parameters*

3D-SIM microscopy was performed on a DeltaVision OMX v4 using an Olympus 60×/1.42 Plan Apo oil objective (Olympus, Japan). The instrument is equipped with 405-, 445-, 488-, 514-, 568-, and 642-nm laser lines (all ≥ 100 mW), and images were recorded on a front-illuminated sCMOS (PCO Photonics, USA) in 286 MHz, 512 × 512 pixel image size mode, 1× binning, 125-nm z-stepping, and with 15 raw images taken per *z* plane (five phase shifts, three angles). A ~10%T laser was used at 5- to 20-ms exposure times. The 0.750- to 1-μm (for mitochondria)– or 2-μm (for lysosomes)–thick z-stacks were recorded for each time point/field of view. Raw image data were computationally reconstructed using CUDA-accelerated 3D-SIM reconstruction code stated above.

### Electron microscopy

HeLa^TMEM192-HA^ control and mutant cells were grown on Aclar plastic coverslips in above stated growth conditions until 70 to 80% confluency was reached, washed twice in 1× PBS, and fixed with a fixation mixture of 2% formaldehyde and 2.5% glutaraldehyde in 0.1 M sodium cacodylate buffer (pH 7.4) for 1 hour at room temperature. Sample preparation and microscopy were performed by the Harvard Medical School Electron Microscopy Facility (https://electron-microscopy.hms.harvard.edu/methods).

### Cryo-plasma FIB–CRYO-ET

#### Cryo-ET sample preparation and freezing

HeLa^TMEM192-HA^ control and *NPC2^−/−^* cells were cultured on EM grids as follows: The 200-mesh gold grids with silicon dioxide R1/4 film (Quantifoil) were plasma-cleaned, coated by incubation with poly-l-lysine (1 mg/ml; Sigma-Aldrich, P2636) solution in 0.1 M borate buffer (pH 8.5 in distilled water, autoclaved) for 2 hours, and washed twice with PBS. One day before plunging, a 150-μl drop of ~150 cells/μl was added on top of each grid and placed in a well of a four-well 35-mm cell culture dish (Greiner Bio-One, 627170); after 2 hours of settling time, DMEM medium was added to a final volume of 2 ml per dish. The next day, cells were starved for 6 hours in phenol red-free EBSS, and 10% glycerol was added to the medium few minutes before plunging. Samples were plunged into ethane/propane with a Vitrobot Mark IV (Thermo Fisher Scientific), with application of 4 μl of EBSS medium and with the following settings: room temperature; humidifier, 70%; blot force, 8; blot time, 9 s. After plunging, the grids were clipped into autogrids with cutout for FIB milling in a custom clipping station (*77*).

#### FIB milling

Transmission electron microscopy (TEM)–transparent lamellae were produced in a commercially available Arctis cryo-PFIB instrument (Thermo Fisher Scientific, Eindhoven, The Netherlands) equipped with a robotic sample delivery device (termed “Autoloader”), CompuStage, NiCol-scanning electron microscope (SEM) column, and Tomahawk FIB column. Preclipped grids were assembled in the standard multispecimen cassette holder so that the cutouts later face the ion beam on the compustage. A xenon ion beam was used for all described steps. After powering on and aligning the beams in the XT software user interface, all consecutive steps were carried out using the proprietary Arctis WebUI software (version 1.0) (*77*, *78*). The grid template sets the parameters for the initial mapping of the grid from the SEM or FIB as well as for the initial protective coating and the final sputter fiducials on the polished lamella. We used here a modified version of the “Electron tileset with auto deposition.” First, a tiled overview perpendicular to the grid was acquired with the SEM with a dwell time of 3 μs and a horizontal field width of 256 μm. Then, points of interest (POIs) were placed at suitable cell positions manually. To protect the leading edge of these positions while milling, a three-step protocol of sputter, chemical vapor, and sputter deposition was carried out. The sputtering process was executed by milling a calibrated regular pattern into an in-built platinum target with a 12-kV xenon beam at a current of 70 nA for 120 s to deposit a thin film of atomic platinum. The platinum fiducials on the end of lamella preparation were induced by the same process but milling only for 5 s. Chemical vapor deposition was executed by heating the attached gas injection system to 28°C and opening the shutter for 50 s to deposit an organo-metallic layer of trimethyl(methylcyclopentadienyl)-platinum(IV) with a thickness of several micrometers on the sample.

The lamella template sets the imaging and milling parameters for the retrieval of the POIs in the ion beam, the milling angle search, the ion beam milling, and the final image acquisition. The set final lamella thickness was refined to 120 ± 10 nm depending on the ice thickness of the respective grid. Briefly, the 30-keV xenon beam milling procedure was as follows: The eucentric height and the maximum milling angle of −18° were refined before milling 0.5-μm-wide stress relief cuts at a distance of 10 μm to each side of the intended lamella using a 1.0-nA ion beam. Three milling steps were then used to remove the material above and below the intended lamella position: (i) rough milling at 3.0 nA to 1-μm thickness, (ii) 0.3 nA to 500 nm, and (iii) 0.1 nA to 300 nm. The respective Silicon depth correction factors were (i) 0.4, (ii) 0.7, and (iii) 0.88. Afterward, the lamella was polished at a current of 30 pA to 110 to 130 nm. In some cases, remnants of the cell top surface with its organometallic layer had to be removed in addition to make the full tilt range in cryo-ET accessible.

#### Cryo-ET data acquisition and processing

TEM data acquisition was performed on a Krios G4 at 300 kV with a Selectris X energy filter and a Falcon 4i camera (Thermo Fisher Scientific, Eindhoven, The Netherlands) using Tomo5 (version 5.12.0, Thermo Fisher Scientific). Tilt series were acquired at a nominal magnification of ×42,000 (pixel size, 2.93 Å) using a dose-symmetric tilt scheme with an angular increment of 2°, a dose of 2 e^−^/Å^2^ per tilt, and a target defocus between −3 and −6 μm. Tilt series were collected ranging from −48° to +60° relative to the lamella pretilt, and frames were saved in the electron-event representation (EER) file format. The positions for tilt series acquisition were determined by visual inspection of ×11,500 magnification “search” montage maps acquired in thin areas of the sample. For publication display, search maps were cleaned and destriped using the Fiji LisC macro, and the contrast was enhanced using contrast-limited adaptive histogram equalization (*79*) (https://github.com/FJBauerlein/LisC_Algorithm; Fig. 4C). Tilt series were acquired of lysosome-like structures. Tilt series frames were motion-corrected with Relion’s implementation of Motioncorr2 (*80*) for EER files. Alignment and contrast transfer function (CTF) correction were performed in IMOD software (v.4.10.49, RRID:SCR_003297, https://bio3d.colorado.edu/imod/) and reconstruction by AreTomo (v.1.3.3) by using an adjusted version of the TomoMAN wrapper scripts (https://doi.org/10.5281/ZENODO.4110737). Tomograms at 2× binning (IMOD bin 4) with a nominal pixel size of 11.72 Å were denoised using cryo-content-aware image restoration (CARE) (https://github.com/juglab/cryoCARE_T2T).

#### Cryo-ET dataset annotation and analysis

Membrane thickness was measured in Gwyddion (*81*) (v.2.63, http://gwyddion.net/) from unbinned, CTF-corrected tomograms after preprocessing with IMOD and EMAN (*82*). To achieve the necessary contrast, tomograms were oriented in the 3Dmod slicer so that the interleaflet space was optimally visible. Tomograms were then rotated, low-pass–filtered to the Nyquist frequency (EMAN2, v.2.99.47, https://blake.bcm.edu/emanwiki/EMAN2), and trimmed to the multilamellar bodies’ volumes. Then, tomograms were averaged in *z* along the slices containing visible membranes and converted to a single 16-bit TIF image. In Gwyddion, the tomogram was inverted, and its minimum intensity value was shifted to zero. Afterward, profiles of similar length were extracted along arbitrary lines perpendicular to the membrane. At least four distinct profiles were placed across the whole membrane, each averaging 64 pixels perpendicular to the drawn line. Afterward, the individual profiles were averaged in OriginLab 2023. Subsequently, peaks were detected, and their FWHM and peak-to-peak distance was analyzed automatically. For attenuated peaks, the peak and the FWHM positions were refined manually. Plots were created in Prism.

#### Tomogram segmentation

All membranes in the tomogram were segmented with Membrain-Seg (https://github.com/teamtomo/membrain-seg) using the publicly available pretrained model (v9), with the exception of the membranes of the MLVs, which were segmented with TomoSegMemTV (*83*) (v.1.0, https://sites.google.com/site/3demimageprocessing/tomosegmemtv) due to their aberrant membrane spacing. All segmentations were then merged and manually refined in Amira.3D (Thermo Fisher Scientific), and final renderings were generated in ChimeraX.
