# Supplementary material for: Global cellular proteo-lipidomic profiling of diverse lysosomal storage disease mutants using nMOST
Source: Sci Adv. 2025 Jan 22;11(4):eadu5787. doi: 10.1126/sciadv.adu5787 (PMC11753374; doi:10.1126/sciadv.adu5787)
Supplement: Supplementary file 1 — Figs. S1 to S7 Legends for tables S1 to S7 Legend for data S1 [file sciadv.adu5787_sm.pdf]

Science Advances
AAAS

Supplementary Materials for

Global cellular proteo-lipidomic profiling of diverse lysosomal storage disease mutants using nMOST

Felix Kraus *et al.*

Corresponding author: Joshua J. Coon, jcoon@chem.wisc.edu; J. Wade Harper, wade_harper@hms.harvard.edu

Sci. Adv. **11**, eadu5787 (2025)
DOI: 10.1126/sciadv.adu5787

The PDF file includes:

Figs. S1 to S7
Legends for tables S1 to S7
Legend for data S1

Other Supplementary Material for this manuscript includes the following:

Tables S1 to S7
Data S1

Supplemental Figure 1

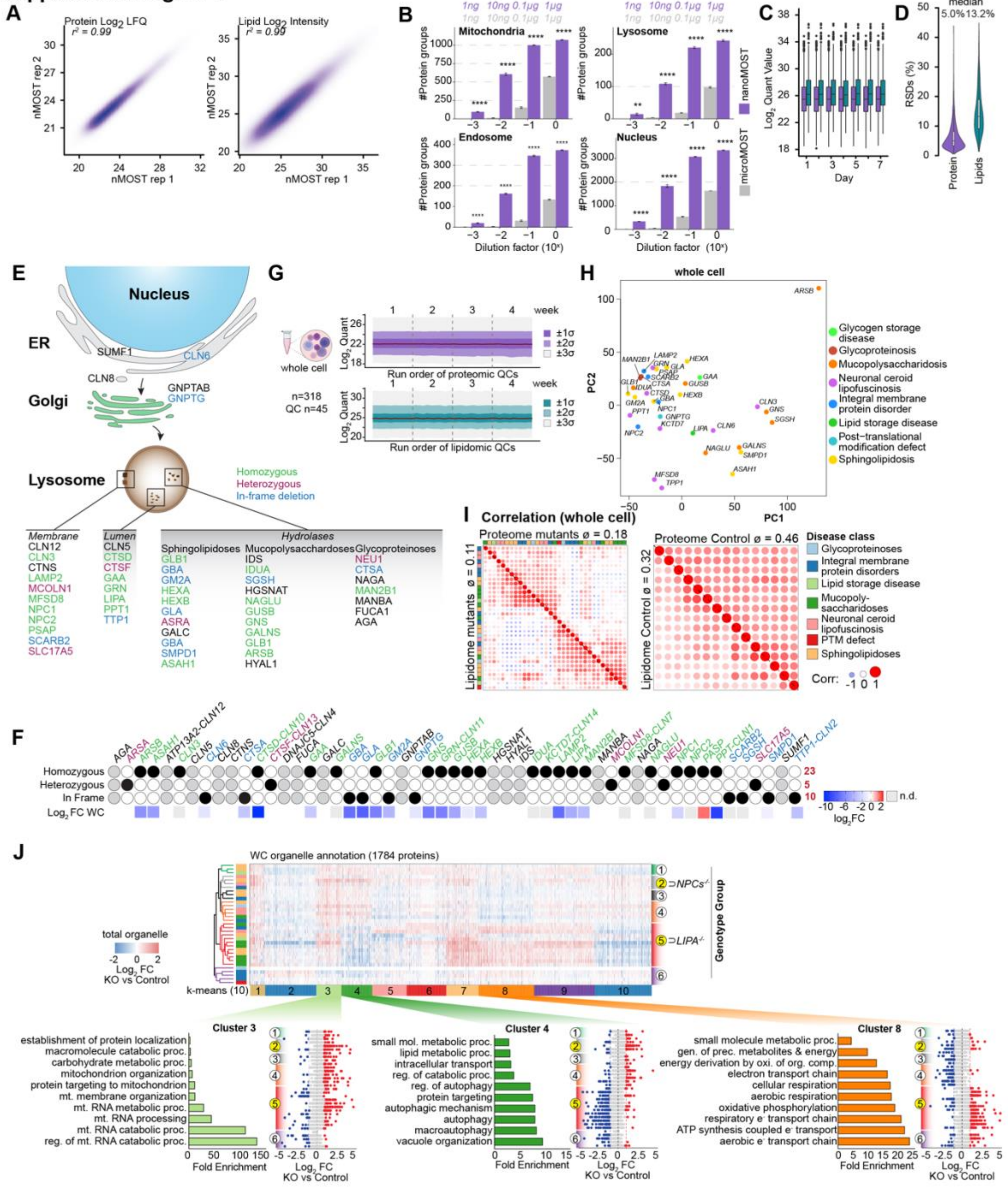

Fig. S1: Benchmarking of nMOST and application to cells lacking LSD genes.

(A) Correlation of $\log_2$ label-free quantification (LFQ) protein (left panel) and lipid biomolecules (right) of two nMOST runs. **(B)** Direct performance comparison of nMOST (purple) with µMOST (grey) over 4 magnitudes of sample dilution. Injection amounts for nMOST and µMOST are listed above. Number of protein groups identified by selected organelles are plotted.

(C) Quantification of number of log_2 quant value (protein and lipid) over a 7-day acquisition period using nMOST. **(D)** Violin plot depicting % relative standard deviations (RSDs) for both quantified protein and lipid identifications over a 7-day acquisition period using nMOST. **(E)** Schematic summarizing 52 LSD proteins and their localization properties when known **(F)** Summary of gene editing champaign with the goal of creating mutants across LSD genes in $HeLa^{TMEM192-HA}$ cells. Black circles indicate the status of mutants obtained. Gray circles indicate no clones for the indicated genes. Lower panel shows log_2FC for all detected LSD proteins in either whole cell extracts from the indicated mutant cell line based on nMOST. **(G)** Log_2 LFQ for total proteomes/lipidomes for 363 total samples analyzed over a 4-week data collection session (318 whole cell extracts with each LSD mutant, untagged Control HeLa and $HeLa^{TMEM192-HA}$ control cells all in quadruplicate biological replicates, and 45 MS QC samples). **(H)** PCA blots for combined proteome and lipidome across the LSD mutants in this study. **(I)** Heatmap depicting correlation of proteome and lipidomes for LSD mutants (top) and controls (bottom). **(J)** Heatmap (log_2FC [Mutant / Control]) of average proteome abundance across the indicated organelle of LSD mutants.

Supplemental Figure 2

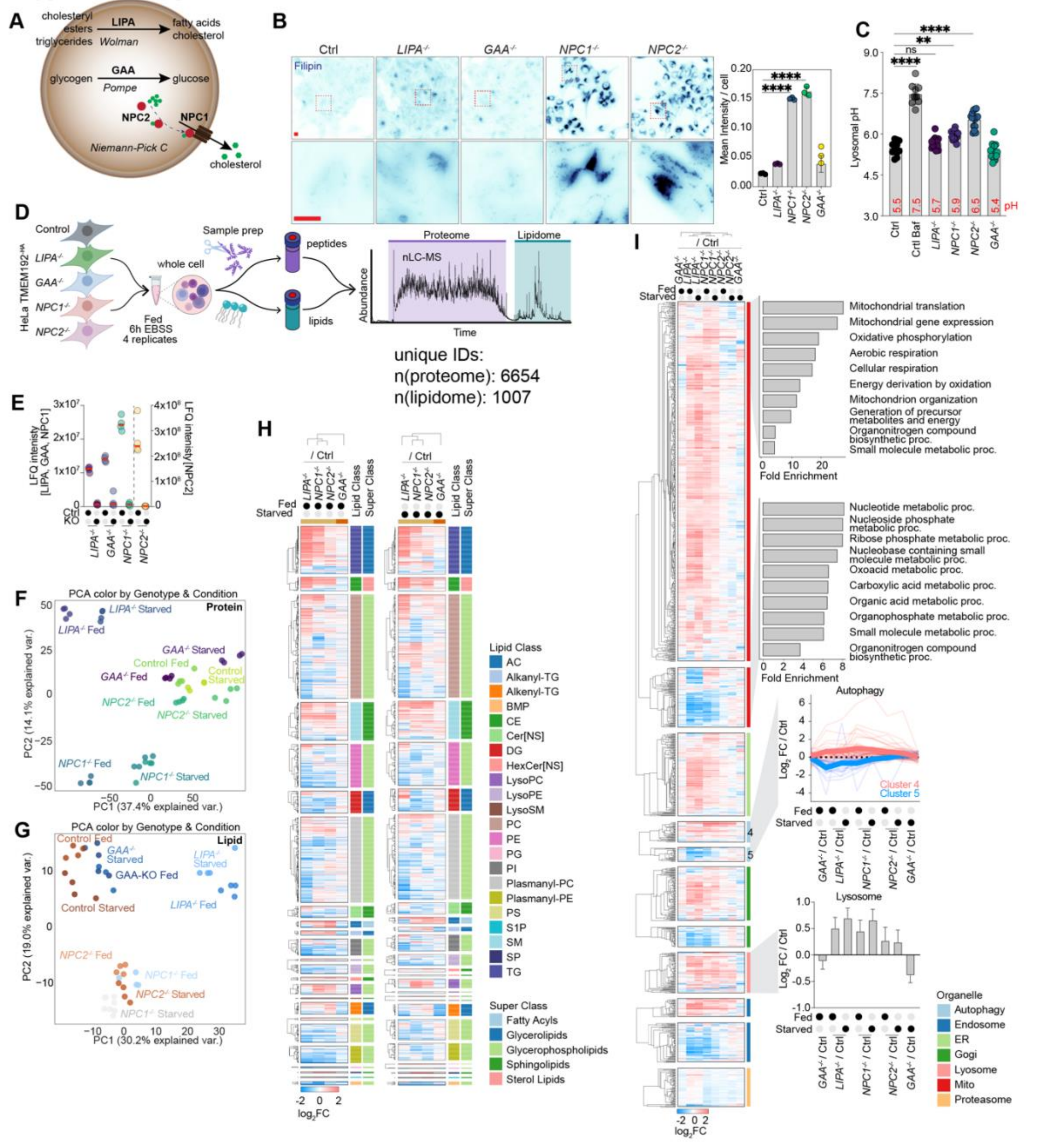

Fig. S2: 4KO-nMOST for profiling autophagy defects in LSDs involved in cholesterol metabolism.

(A) The general functions of the four proteins selected for 4KO study (*LIPA*$^{-/-}$, *GAA*$^{-/-}$, *NPC1*$^{-/-}$ and *NPC2*$^{-/-}$) within the lysosome is shown in the schematic. **(B)** Wide-field fluorescent images of Hela Control and 4KO cell lines stained for cholesterol with Filipin. Quantification of mean Filipin intensity per cell is plotted below (data from three biological replicates, 20 image stacks

per repeat; genotype(N)): Ctrl(1424), *LIPA*$^{-/-}$(2123), *GAA*$^{-/-}$ (1791), *NPC1*$^{-/-}$ (1773), *NPC2*$^{-/-}$ (3270)). p(****) <0.0001, ordinary two-way ANOVA with multiple comparisons, alpha = 0.05; error bars depict S.D. Scale bar = 20 µm. **(C)** pH measurements for 4KO cells using ratiometic confocal imaging. Each data point represents one field of view; Repeats per genotype (N): Ctrl(12), Ctrl + BafA(10), *LIPA*$^{-/-}$, *GAA*$^{-/-}$, *NPC1*$^{-/-}$, *NPC2*$^{-/-}$(15). p(****) <0.0001; p(**) = 0.0022; ordinary one-way ANOVA with multiple comparisons, alpha = 0.05; error bars depict S.D. **(D)** Application of nMOST for analysis of Control, *LIPA*$^{-/-}$, *GAA*$^{-/-}$, *NPC1*$^{-/-}$ and *NPC2*$^{-/-}$ cells (4KO cells). The 5 indicated HeLa$^{TMEM192\text{-}HA}$ cell lines were analysed in quadupliates for both fed and starvation conditions. Number of unique IDs for proteins and lipids are shown under the chromatograph. **(E)** LFQ of LIPA, GAA, NPC1 and NPC2 in Control and mutant cells based on nMOST data. Data based on quadruplicate replicate nMOST measurements. **(F,G)** PCA analysis of 4KO proteomic (panel A) and lipidomic (panel B) data from nMOST analysis of the indicated cell lines under Fed or EBSS (6 hoursours) conditions. Data based on quadruplicate biological replicate nMOST measurements. **(H)** Heatmap of $\log_2$ abundance of lipids under indicated treatment conditions. Lipid classes / super classes are highlighted on the right of the heatmap. Data based on quadruplicate biological replicate nMOST measurements. **(I)** Heatmap of $\log_2$ abundance of organelle-annotated proteins under indicated treatment conditions. Top two graphs show results of GO-term enrichment analysis associated with the two mitochondrial clusters. Abundance for autophagy clusters 4 and 5 are plotted on the right. Abundance of lysosome is plotted at the bottom right bar-graph. Data based on quadruplicate biological replicate nMOST measurements.

Supplemental Figure 3

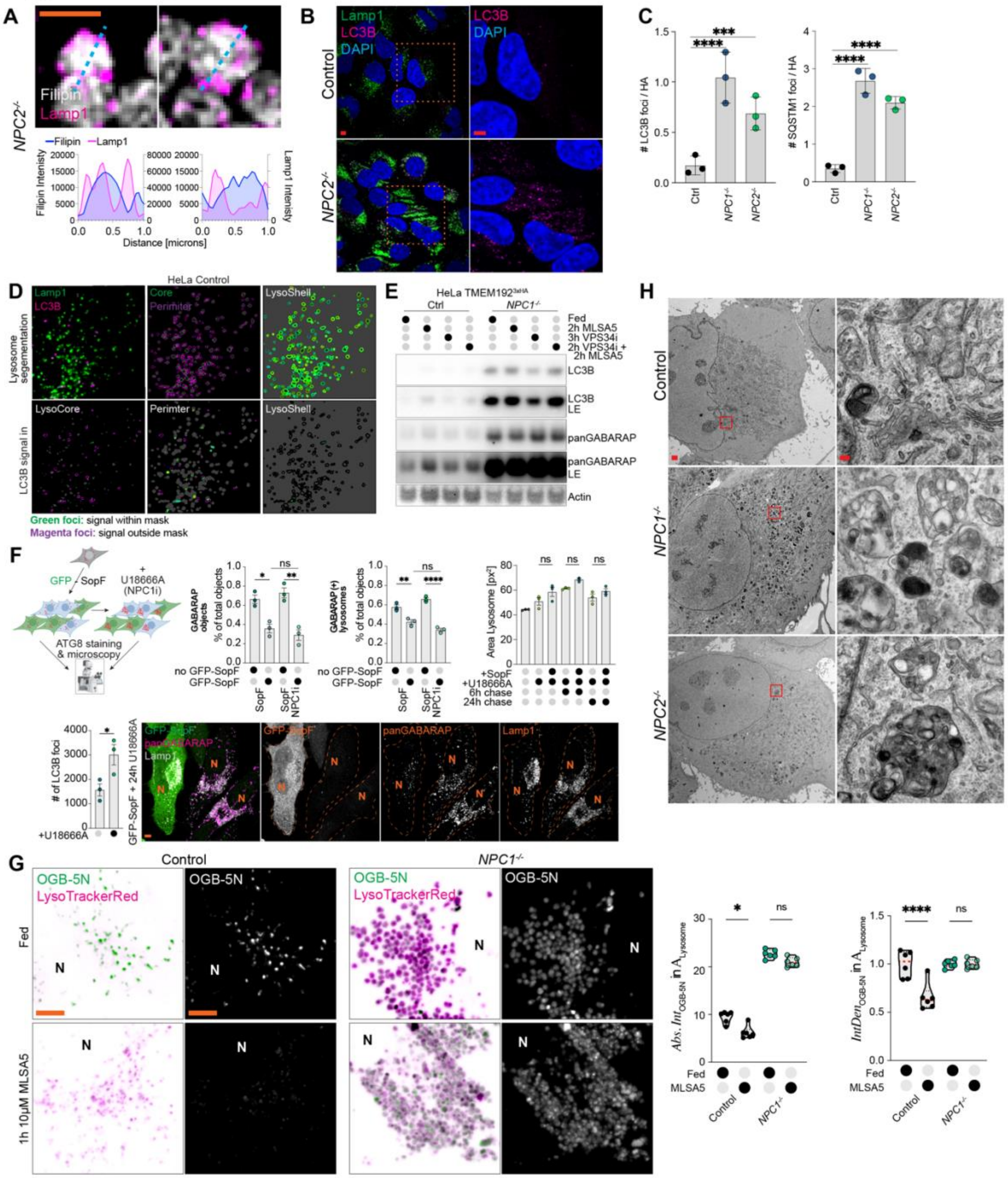

Fig. S3: Profiling of lysosomal function in NPC1 and NPC2 mutant cells.

(**A**) Example of Filipin-positive lysosomes and a-LAMP1 signal derived from 3D-SIM imaging. Scale bar = 1 µm. Lineplot of filipin and LAMP1 intensities for the dashed line are plotted beneath.

(B) Immunostaining of Control and *NPC2*$^{-/-}$ cells with a-LAMP1 and a-LC3B. Nuclei were stained with DAPI. Scale bars = 5 µm. **(C)** Evaluation based on confocal imaging of Control, *NPC1*$^{-/-}$ and *NPC2*$^{-/-}$ cells immunostained with a-LC3, a-SQSTM1, and a-HA to detect TMEM192^{-HA}. Quantification was performed on three biological replicates with 5 stacks in each replicate. MAPLC3B: p(****) <0.0001; p(***) = 0.0002; p(*)=0.0129 & 0.0157. p62/SQSTM1: p(****) <0.0001. Data from quadruplicate replicates, ordinary one-way ANOVA with multiple comparisons, alpha = 0.05. Error bars depict S.D. (**D**) Example for segmentation and analysis strategy for quantifying LC3B localization relative to LAMP1. Input image is segmented and filtered to create a lysosomal core & perimeter mask, and the resulting lysosomal shell (Perimeter \ Core). Underneath, example results for LC3B signal in the different localization in a HeLa Control cell is shown. Green foci depict LC3B signal that reside within the specific mask, magenta-coloured foci represent foci that are outside the specific mask. **(E)** Western Blot for select autophagy proteins of whole cell lysates from HeLa Control and *NPC1*$^{-/-}$ mutants treated with MLSA5 and/or VPS34inhibitor. **(F)** Schematic of experimental approach to study role of GFP-SopF and lysosomal ATG8lyation in relationship to NPC1 inhibition. Example confocal images of GFP-SopF expressing Hela cells immunostained for a-LAMP1 and a-panGABARAP. Scale bar = 5 µm. GABARAP per cell comparisons: A-B: p(*) = 0.0103. C-D: p(**) = 0.0011. GABARAP per lysosome comparisons: A-B: p(**) = 0.0033. C-D: p(****) = <0.0001. LC3B p(*) = 0.0180. Data from 3 replicates with 15 stacks each. One-way ANOVA with multiple comparisons, alpha = 0.05. Error bars depict S.E.M. **(G)** Images from live-cell microscopy of Control and *NPC1*$^{-/-}$ in fed and MLSA5-treated conditions. Lysosomes are stained with LysoTrackerRed and lysosomal Ca^{2+} using OGB-5N. Scale bar = 5 µm. Violin plots of quantification of lysosomal Ca^{2+} intensity ± MLSA5 treatment in absolute measures (left) and relative to lysosomal area (right). Quantification was performed on five replicates with 3 image stacks in each replicate. Ctrl: p(****) <0.0001; p(*)=0.0143. Two-way ANOVA with multiple comparisons, alpha = 0.05. **(H)** Control, *NPC1*$^{-/-}$ or *NPC2*$^{-/-}$ cells were examined by electron microscopy. Scale bars = 1 µm (left & middle panel) and 100 nm (right panel).

Supplemental Figure 4

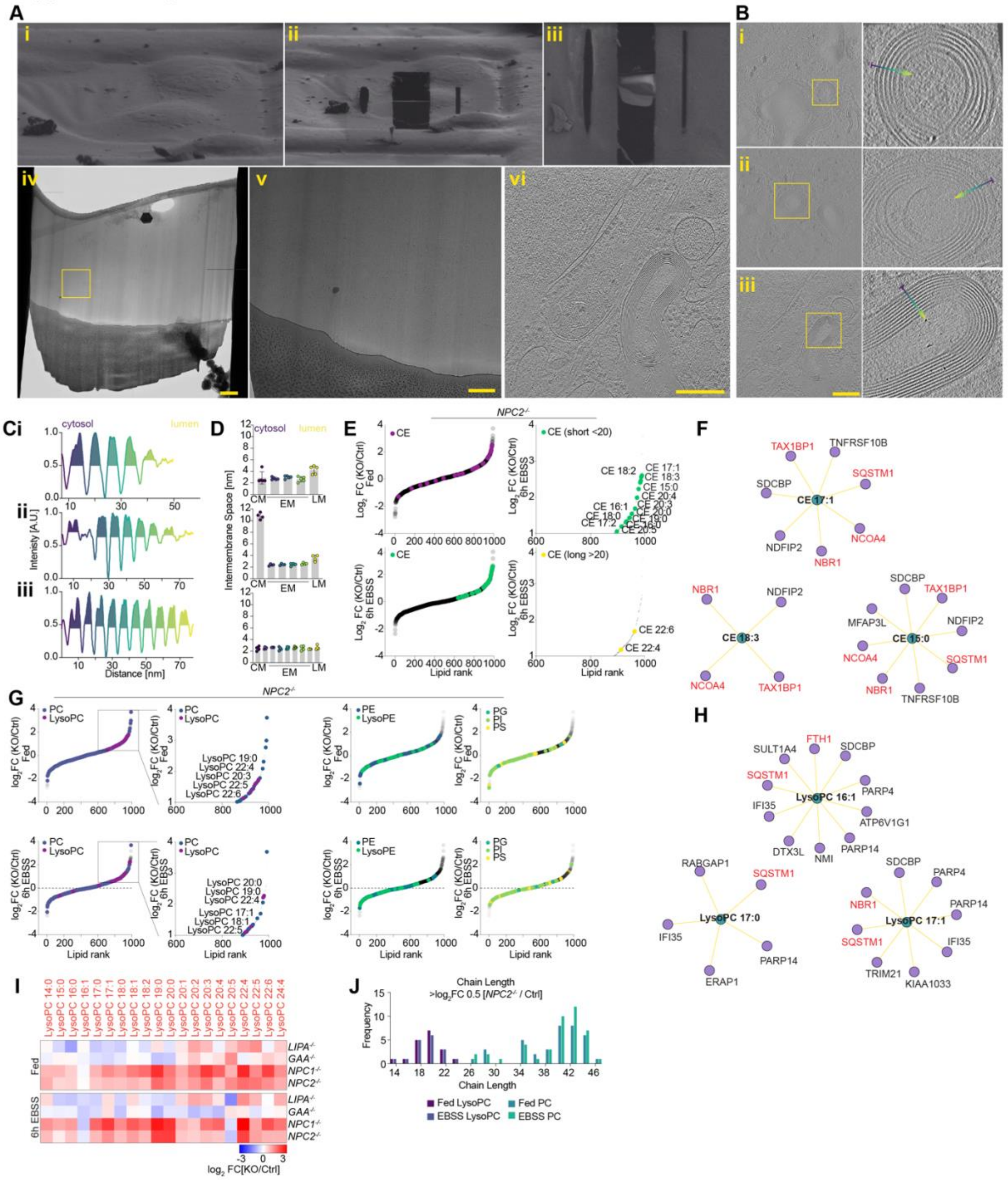

Fig. S4. Visualization of multi-lamellar membranes in *NPC2*$^{-/-}$ lysosomes by cryo-ET.

(A) Example images of the cryo-PFIB and cryo-ET workflow. Vitrified cells before (i) and after (ii) lamella preparation by cryo-PFIB milling. (iii) final SEM view of milled and polished lamella. (iv-v) Lamella overview with zoom-in on a MLV containing area. (vi) Reconstructed

tomogram showing an MLV. Scale: (i-ii) 156 µm horizontal field width (HFW), (iii) 124 µm HFW. Scale bar = (iv) 1 µm (v) 500 nm (vi) 250 nm. (**B**) Overview images and zoom-ins of three tomograms depicting MLV membrane stacks. Scale bar = 250 nm. **(C)** Averaged, inverted intensity along the arrows from B to determine membrane thickness. The gradient indicates the measurement direction from cytosol (purple) to lumen (yellow). Membrane peaks are coloured to indicate their full width at half maximum. **(D)** Intermembrane Space of selected MLVs between adjacent membrane pairs. The gradient indicates the measurement direction from cytosol (purple) to lumen (yellow). **(E)** Ranked lipid log_2FC abundance of *NPC2*$^{-/-}$ lipidome for fed and 6 hours EBSS nutrient starvation conditions. Cholesterol esters (CE) are highlighted in colour on top of the overall lipidome spread. The lower row depicts ranking of short or longer CE in 6 hours EBSS nutrient starvation conditions against the whole lipidome rank. Data based on quadruplicate biological replicate nMOST measurements. **(F)** Lipid-protein networks of select CE species based on cross-ome correlations of the LSD-nMOST dataset. **(G)** Ranked lipid log_2FC abundance of phospholipids in *NPC2*$^{-/-}$ lipidome for fed and 6 hours EBSS nutrient starvation conditions. Lysosomal specific phospholipids (LysoPC/LysoPE) are highlighted in color on top of the overall lipidome and parent-lipid class spread. Lower row depicts the lipidome of annotated lipids in 6 hours EBSS nutrient starvation conditions against the whole lipidome rank. Data based on quadruplicate biological replicate nMOST measurements. **(H)** Lipid-protein networks of select LysoPC species based on cross-ome correlations of the LSD-nMOST dataset. **(I)** Heatmap depicting log_2FC of LysoPC species in either Fed and EBSS-treated Control and 4KO cells. Data based on quadruplicate biological replicate nMOST measurements. **(J)** Histogram depicting frequency of (Lyso-)PCs enriched ≥ 0.5 log_2FC in [*NPC2*$^{-/-}$/Control] against their chain length. Data based on quadruplicate biological replicate nMOST measurements.

Supplemental Figure 5

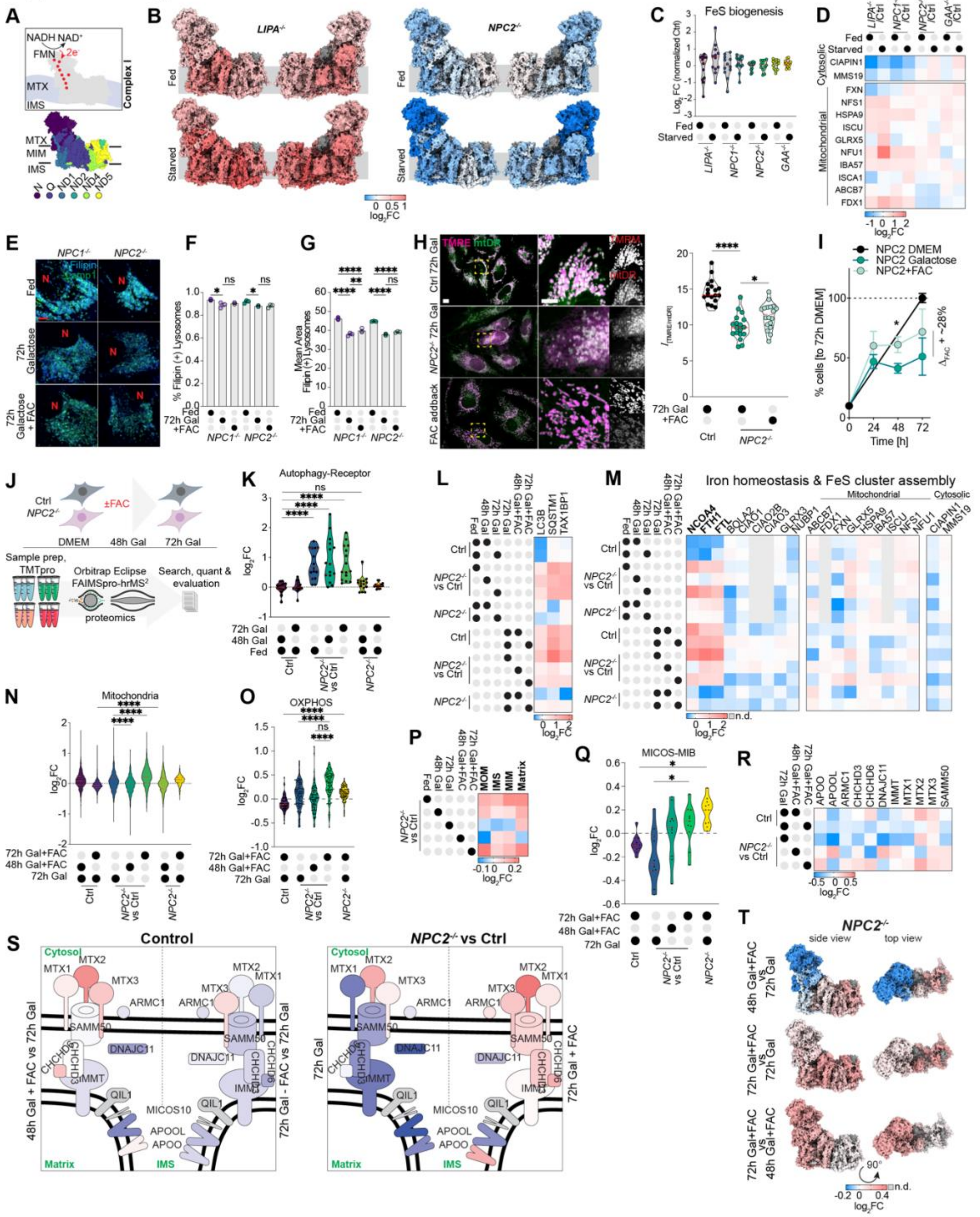

Fig. S5. Profiling of mitochondrial proteome in 4KOs and alleviation of mitochondrial defects in *NPC2*$^{-/-}$ cells by extracellular iron.

(A) Schematic of CI of the OXPHOS system with individual sub-modules (PDB: 5XTH). **(B)** Log_2FC of CI sub-module abundance in Fed and EBSS-treated conditions measured in *LIPA*$^{-/-}$,

and *NPC2*$^{-/-}$ cells by nMOST [normalized to control]. Based on quadruplicate replicate nMOST data. Legend shows colour panel for log_2FC values. **(C,D)** Log_2FC Violinplot and heatmap for components of the mitochondrial and cytosolic FeS cluster biogenesis system for 4KO cells in Fed and EBSS-treated cells based on quadruplicate replicate nMOST data. **(E)** Confocal images of HeLa *NPC1*$^{-/-}$ and *NPC2*$^{-/-}$ in indicated growth media conditions immunostained with a-LAMP1 and cholesterol-rich lysosomes were stained with Filipin. Scale bar = 5 µm. **(F)** Quantification of % Filipin(+) lysosomes in different growth media conditions. Data based on three biological replicates with 6 image stack per repeat. *NPC1*$^{-/-}$: p(*) = 0.03, *NPC2*$^{-/-}$: p(*) = 0.0124. Unpaired t-test. Error bars depict S.E.M.. **(G)** Quantification of mean lysosomal size in different growth media conditions. Data based on three biological replicates with 6 image stack per repeat. *NPC1*$^{-/-}$: A-B: p(****) = <0.0001, A-C: p(****) = <0.0001, B-C: : p(**) = 0.0021. *NPC2*$^{-/-}$: A-B: p(****) = <0.0001, A-C: p(****) = <0.0001. Two-way ANOVA with multiple comparisons, alpha = 0.05. Error bars depict S.E.M.. **(H)** Stills of spinning-disk live-cell microscopy of Control and *NPC2*$^{-/-}$ cells cultured in Galactose for 72 hours or in Galactose for 72 hours with FAC. Mitochondria are stained with TMRE ($\Delta\Psi$m) and MitoTrackerDeepRed. Scale bar = 10 µm and 5 µm (insets). Right panel shows quantification of $I_{[mtDR\text{-}TMRE]}$ for indicated genotypes and treatments. Data from four biological replicates (18 stacks per replicates); p(****) < 0.0001, p(*) = 0.0150; unpaired t.test. **(I)** % of cell count of *NPC2*$^{-/-}$ [relative to growth in 72 hours DMEM] in Galactose ± FAC over a 72 hours growth period. **(J)** Schematic of TMT proteomics workflow for analysis of the effect of FAC addition to Control or *NPC2*$^{-/-}$ cells. **(K,L)** Violin plot of all autophagy receptors (panel K) and heatmap of LC3B, SQSTM1 and TAX1BP1 (panel L) for log_2FC [*NPC2*$^{-/-}$/Control] in cells cultured in Galactose in the presence or absence of FAC. p(****) < 0.0001; data based on biological triplicate TMTpro measurements. **(M)** Heatmap of log_2FC [*NPC2*$^{-/-}$/Control] for components of the cytosolic and mitochondrial FeS cluster assembly system as well as the Ferritin system with or without FAC with cells grown in Galactose. Data based on biological triplicate TMTpro measurements. **(N)** Violin plots for total mitochondrial proteins in Control versus *NPC2*$^{-/-}$ cells grown in Galactose ± FAC. p(****) < 0.0001; data based on biological triplicate TMTpro measurements. **(O)** Violin plot of OXPHOS subunit log_2FC values in *NPC2*$^{-/-}$ versus Control cells grown in Galactose with or without FAC. p(****) < 0.0001, ordinary one-way ANOVA with multiple comparisons, alpha = 0.05; data based on triplicate biological replicate TMTpro measurements. **(P)** Heatmap

depicting log_2 FC of components of different mitochondrial compartments in cells cultured in Glucose and Galactose in the presence or absence of FAC. Data based on biological triplicate TMTpro measurements. **(Q)** Violin plots of log_2FC [*NPC2*$^{-/-}$/Control] of MICOS-MIB subunits in response to FAC. Data based on biological triplicate replicate TMTpro measurements; p(*) = 0.0162. **(R)** Heatmap of log_2FC [*NPC2*$^{-/-}$/Control] for individual MICOS-MIB subunits in response to FAC. Data based on triplicate biological replicate TMTpro measurements. **(S)** Schematic showing alterations in various MICOS-MIB subunits in response to FAC. Colour coding is based on log_2FC scale in panel R. **(T)** Log_2FC of CI abundance in Galactose with or without FAC addback for *NPC2*$^{-/-}$ cells. Legend shows color panel for log_2FC values. Data based on triplicate biological replicate TMTpro measurements.

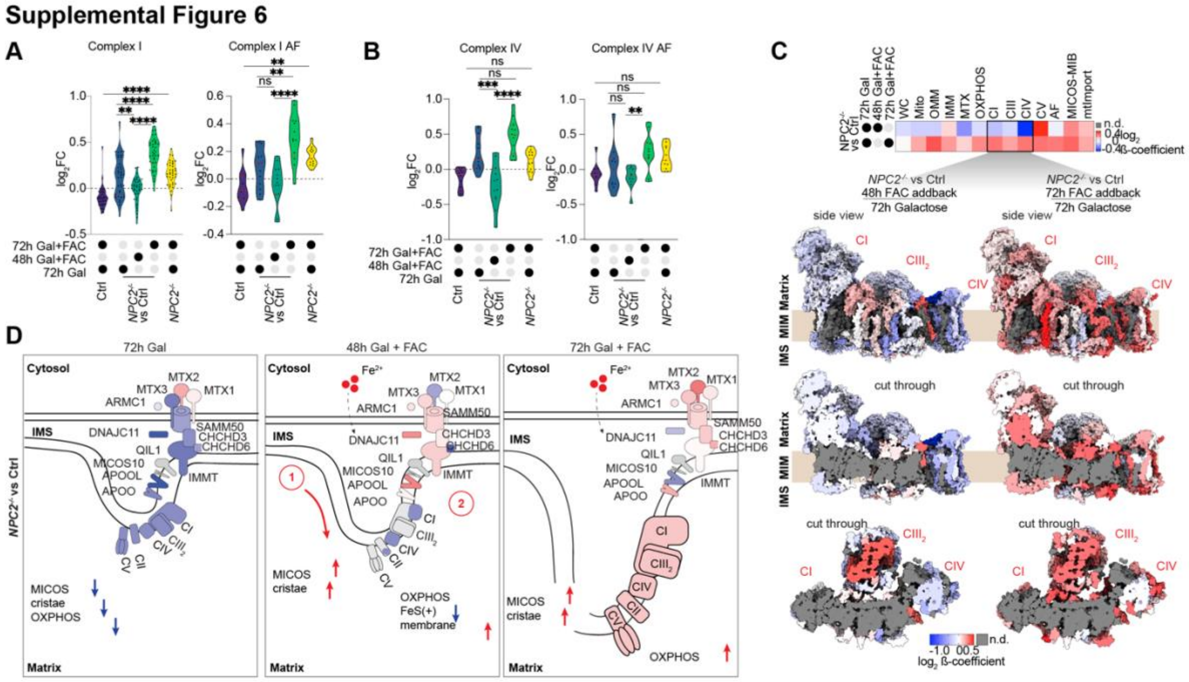

Fig. S6. Rescue of OXPHOS complex abundance in *NPC2*$^{-/-}$ cells by extracellular iron. **(A,B)** Violin plots of log_2FC values for CI (panel D) and CIV (panel E) subunits (left panels) and associated assembly factors (right panels) in *NPC2*$^{-/-}$ versus Control cells grown in Galactose with or without FAC. Complex I: p(****) < 0.0001, p(**) = 0.0036; Complex I AF: p(****) < 0.0001, p(**) = 0.0018; Complex IV: p(****) < 0.0001, p(***) = 0.0002; Complex IV AF: p(**) = 0.0056; ordinary one-way ANOVA with multiple comparisons, alpha = 0.05; data based on biological triplicate TMTpro measurements. **(C)** Log_2FC of β-coefficient of mitochondrial components (see middle heatmap) in Galactose and either 48 or 72 hours FAC addback for

NPC2$^{-/-}$ versus Control cells. Abundance of supercomplex subunits is mapped onto the structure (PDB: 5XTH). Vertical and horizontal cut throughs of the structure are depicted in the lower panels. Legend shows color panel for log_2FC values. Data based on biological triplicate TMTpro measurements. **(D)** Schematic model for the rescue of mitochondrial cristae and OXPHOS complexes upon FAC addback in *NPC2*$^{-/-}$ cells. See text for details.

Supplemental Figure 7

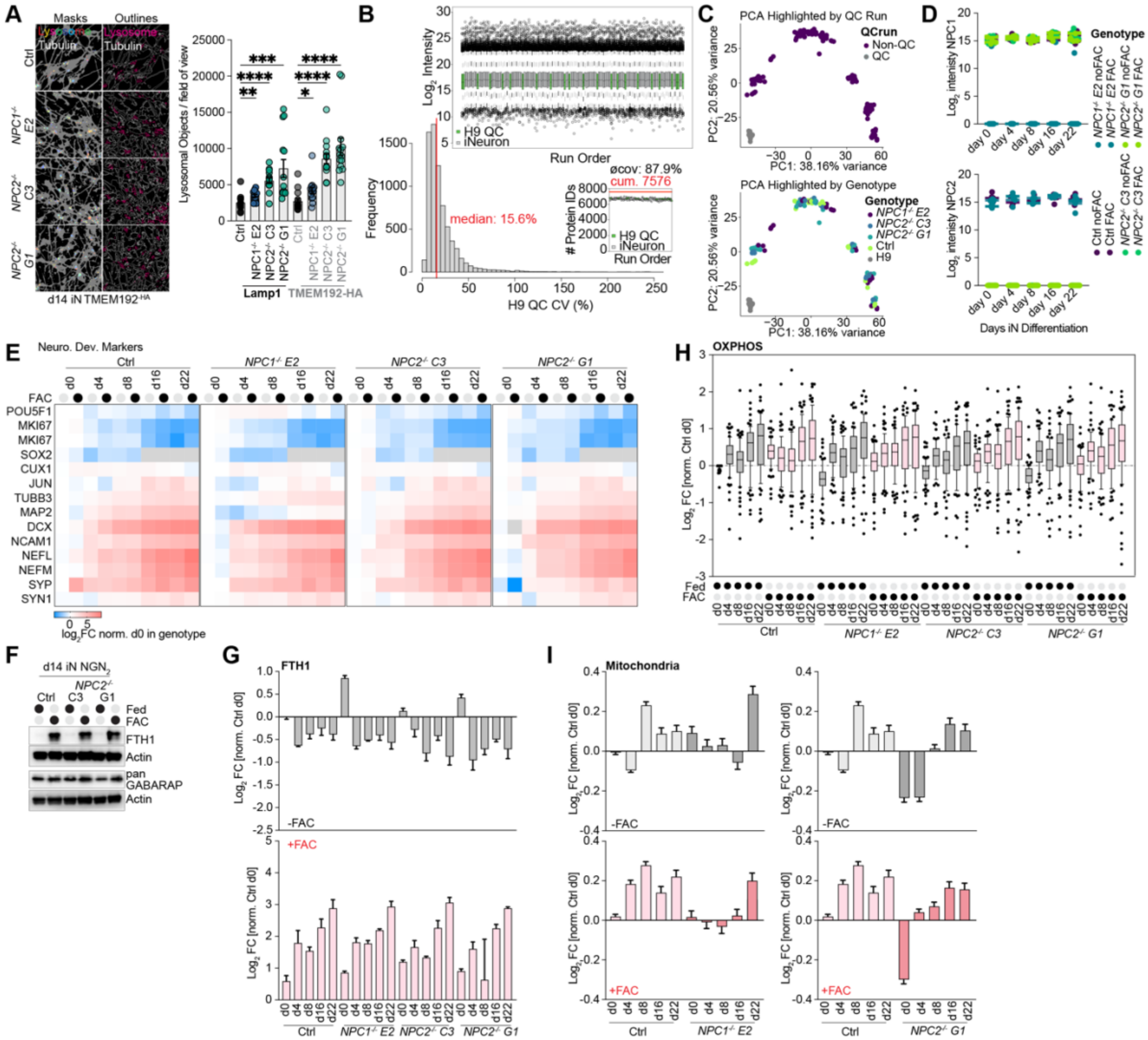

Fig. S7. Neuronal proteomics of NPC mutants in presence of FAC.

(A) Example object segmentation overlays (lysosome & tubulin) of day 14 iNeurons of the indicated genotypes. Quantification of lysosomal objects per stack for both a-LAMP1 and a-HA. Unpaired t.test Lamp1: *NPC1*$^{-/-}$ E2: p(**) = 0.0079, *NPC2*$^{-/-}$ C3: p(****) <0.0001, *NPC2*$^{-/-}$ G1: p(***) = 0.0009. HA: *NPC1*$^{-/-}$ E2: p(*) = 0.0167, *NPC2*$^{-/-}$ C3: p(****) <0.0001, *NPC2*$^{-/-}$ G1: p(****) < 0.0001. Data based 14 replicates. Error bars show S.E.M.. **(B)** QC-assessment of LFQ proteome data from iNeuron ± FAC. Boxplot of $\log_2$ intensity across the 142 LC-MS runs. Green boxplots depict H9 ESC QC samples, grey boxplots depict time-course samples (d0 – d22). Frequency distribution of H9 QC sample coefficient of variation (CV) across the 22 H9 ESC QC

samples, covering the whole acquisition time-window. Average unique protein group coverage rate per run. On average 6659 protein IDs were detected. **(C)** PCA plot of LFQ data, color-coded according to run or genotype. **(D)** Log_2 intensity for NPC1 (top) or NPC2 (bottom) across all time-points for the indicated genotypes. **(E)** Heatmap of neuronal development markers (log_2FC norm. within genotype) across all time-points for the indicated genotypes. **(F)** Western blot of FTH1 and panGABARAP from iNeurons at day 14 of differentiation of Control and two *NPC2*$^{-/-}$ clones ± FAC. **(G)** Bargraph of mean log_2FC (normalized within genotype day 0) of FTH1 ± FAC across all time-points and genotypes. **(H)** Boxplot of log_2FC (normalized to Control at day 0) mitochondrial OXPHOS-components across the indicated genotypes, differentiation times and ± FAC treatment. **(I)** Bargraph of mean log_2FC (normalized within genotype day 0) of the mitochondrial proteome ± FAC across all time-points.

Table S1. Generation of CRISPR-edited cell lines for interrogation of lysosomal storage disease gene function analysis. This file contains gRNA sequences as well as allele sequencing results for all edits examined. Relevant to **Figure S1E, S1F**.

Table S2. nMOST proteomic and lipidomic analysis of 33 LSD cell lines (total proteome). Related to **Figure 2, Figure 3, S1G-I.**

Table S3. nMOST Cross-Ome analysis of 33 LSD cell lines (whole cell). Related to **Figure 2, 3A-C.**

Table S4. nMOST proteomic and lipidomic analysis of Control, *LIPA*$^{-/-}$, *GAA*$^{-/-}$, *NPC1*$^{-/-}$ and *NPC2*$^{-/-}$ HeLa$^{TMEM192\text{-}HA}$ cells under fed and starved (EBSS) conditions. Related to **Figure 4A, S2D-I, S4E-H, 5A-E, S5A-D**.

Table S5. TMTpro-based proteomic analysis of Control and *NPC2*$^{-/-}$ HeLa$^{TMEM192\text{-}HA}$ with and without FAC addition. Relevant to **Figure 5L, M; S5J-T, S6A-D.**

Table S6. nDIA label-free whole cell proteomics of neuronal differentiation timecourse (d0, d4, d8, d16, d22) of H9$^{TMEM192\text{-}HA}$ NGN2 Control, *NPC1*$^{-/-}$ *E2*, *NPC2*$^{-/-}$ *C3*, *NPC2*$^{-/-}$ *G1*. Relevant to **Figure 7, S7B-E, H-I.**

Table S7. Source data file containing data tabulated data used in figures.

Data S1. (separate file)
Key Resource Table
